# A High-Density Linkage Map of the Forage Grass *Eragrostis curvula* and Localization of the Diplospory Locus

**DOI:** 10.3389/fpls.2019.00918

**Published:** 2019-07-12

**Authors:** Diego Zappacosta, Jimena Gallardo, José Carballo, Mauro Meier, Juan Manuel Rodrigo, Cristian A. Gallo, Juan Pablo Selva, Juliana Stein, Juan Pablo A. Ortiz, Emidio Albertini, Viviana Echenique

**Affiliations:** ^1^Departamento de Agronomía, Centro de Recursos Naturales Renovables de la Zona Semiárida (CERZOS-CONICET, CCT Bahía Blanca), Universidad Nacional del Sur, Bahía Blanca, Argentina; ^2^Laboratorio Biotecnológico, Asociación de Cooperativas Argentinas Coop. Ltd., Pergamino, Argentina; ^3^Laboratorio de Biología Molecular, Facultad de Ciencias Agrarias, Universidad Nacional de Rosario, Instituto de Investigaciones en Ciencias Agrarias de Rosario (IICAR, CONICET-UNR), Zavalla, Argentina; ^4^Dipartimento di Scienze Agrarie, Alimentari e Ambientali, Università degli Studi di Perugia, Perugia, Italy

**Keywords:** linkage map, weeping lovegrass, apomixis, polyploid, QTL, synteny

## Abstract

*Eragrostis curvula* (Schrad.) Nees (weeping lovegrass) is an apomictic species native to Southern Africa that is used as forage grass in semiarid regions of Argentina. Apomixis is a mechanism for clonal propagation through seeds that involves the avoidance of meiosis to generate an unreduced embryo sac (apomeiosis), parthenogenesis, and viable endosperm formation in a fertilization-dependent or -independent manner. Here, we constructed the first saturated linkage map of tetraploid *E. curvula* using both traditional (AFLP and SSR) and high-throughput molecular markers (GBS-SNP) and identified the locus controlling diplospory. We also identified putative regulatory regions affecting the expressivity of this trait and syntenic relationships with genomes of other grass species. We obtained a tetraploid mapping population from a cross between a full sexual genotype (OTA-S) with a facultative apomictic individual of cv. Don Walter. Phenotypic characterization of F_1_ hybrids by cytoembryological analysis yielded a 1:1 ratio of apomictic vs. sexual plants (34:27, *X*^2^ = 0.37), which agrees with the model of inheritance of a single dominant genetic factor. The final number of markers was 1,114 for OTA-S and 2,019 for Don Walter. These markers were distributed into 40 linkage groups per parental genotype, which is consistent with the number of *E. curvula* chromosomes (containing 2 to 123 markers per linkage group). The total length of the OTA-S map was 1,335 cM, with an average marker density of 1.22 cM per marker. The Don Walter map was 1,976.2 cM, with an average marker density of 0.98 cM/marker. The locus responsible for diplospory was mapped on Don Walter linkage group 3, with other 65 markers. QTL analyses of the expressivity of diplospory in the F_1_ hybrids revealed the presence of two main QTLs, located 3.27 and 15 cM from the diplospory locus. Both QTLs explained 28.6% of phenotypic variation. Syntenic analysis allowed us to establish the groups of homologs/homeologs for each linkage map. The genetic linkage map reported in this study, the first such map for *E. curvula*, is the most saturated map for the genus *Eragrostis* and one of the most saturated maps for a polyploid forage grass species.

## Introduction

Apomixis, a clonal mode of reproduction through seeds, occurs in numerous plant families and in organisms from other kingdoms (Asker and Jerling, 1992). Apomixis can be divided in two main types based on the origin of the clonal embryos. During adventitious embryony or sporophytic apomixis, the embryo develops directly from a somatic cell in the ovule (usually the nucellus or integument) outside of the sexual embryo sac. Survival of the apomictic embryo depends on the successful fertilization of the meiotically derived embryo sac and its ability to grow sufficiently to gain access to the endosperm (Koltunow and Grossniklaus, 2003). During gametophytic apomixis, unreduced embryo sacs form by mitosis of a megaspore mother cell that avoids meiosis (diplospory) or via a mitotic division of a nucellar cell (apospory) (Koltunow and Grossniklaus, 2003). The embryo then forms by fertilization-independent embryogenesis (parthenogenesis), and the endosperm develops autonomously or after fertilization of the polar nuclei (pseudogamy) (Koltunow et al., 2013). This mode of reproduction is present in more than 400 plant species, representing approximately 40 families. The occurrence of adventitious embryony has been reported in 148 genera, apospory has been reported in 110 genera, and diplospory has been reported in 68 genera (Hojsgaard et al., 2014). The apomictic trait has a polyphyletic origin, and the genes and mechanisms involved in its expression and regulation are diverse. Therefore, research on this reproductive mechanism should focus specifically on each apomictic species (Crane, 2001).

Apomixis has great potential for enhancing plant breeding and seed production, as it enables the fixation and unlimited propagation of complex, heterozygous genotypes (Spillane et al., 2001). Despite the efforts that have been made toward transferring this trait to crop species using various approaches, such attempts have thus far been unsuccessful (Kandemir and Saygili, 2014). Although transgenesis appears to be an optimum way to transfer this trait to crops, it is imperative to determine the molecular pathways and genes responsible for apomixis. Several strategies have been used to gain insight into the genetic basis of apomixis, including interspecific hybridizations between sexual crops and apomictic wild relatives (Savidan et al., 2001), unraveling its genetic control in natural apomicts (Albertini et al., 2005; Corral et al., 2013; Siena et al., 2014; Conner et al., 2015; Pellegrini, 2016; Garbus et al., 2017; Selva et al., 2017), identifying mutants of sexual species that mimic apomixis components (Garcia-Aguilar et al., 2010; Olmedo-Monfil et al., 2010), and recreating an apomictic phenotype in a sexual background (Ravi et al., 2008; d’Erfurth et al., 2009).

Analyses of segregating populations derived from crosses between sexual (as the female parent) and apomictic (as the pollen donor) genotypes have led to the development of several models to explain the inheritance of the trait and its components (apomeiosis and parthenogenesis) (Ozias-Akins and van Dijk, 2007). The proposed mechanisms differ in terms of the number and functions of genes and allelic relationships, as well as the effects of dominance over sexuality (Carman, 1997; Grimanelli et al., 2001; Koltunow and Grossniklaus, 2003). Nevertheless, most of these studies agree that one or a few Mendelian factors control the transference and expression of apomeiosis and its components in most species (Ozias-Akins and van Dijk, 2007; Pupilli and Barcaccia, 2012). By contrast, molecular and cytogenetic analyses suggest that this trait is controlled in a complex genetic manner involving restricted recombination around the apomixis locus, *trans-*elimination of gametes, supernumerary chromatic structures, DNA rearrangements, and the presence of transposons in several species (reviewed by Albertini et al., 2010 and Ortiz et al., 2013). These characteristics have been the main drawback preventing the isolation of apomixis determinants in natural species.

*Eragrostis curvula* (Schrad.) Nees (weeping lovegrass) is a pseudogamous apomictic grass species native to Southern Africa (Streetman, 1963) that is used as forage in the United States, Australia and Argentina. The *E. curvula* complex includes cytotypes with different ploidy levels (e.g., 2×–8×) displaying obligate apomixis, facultative apomixis, and sexual reproduction (Voigt and Bashaw, 1976; Voigt et al., 2004). This grass is considered to be an allopolyploid species, although multivalent formation has been recorded in some polyploid genotypes (Vorster and Liebenberg, 1977, Poverene, 1988). Moreover, Burson and Voigt (1996) have shown that this grass behaves as segmental allotetraploid.

The genus *Eragrostis* has a unique diplosporous apomictic type (*Eragrostis* type) characterized by the lack of meiotic stages in which the megaspore mother cell undergoes two rounds of mitotic division, leading to the formation of an unreduced 4-nucleate embryo sac containing an egg, two synergids, and one polar nucleus (Meier et al., 2011). Following parthenogenetic development of the unreduced egg cell to form a maternal embryo, the endosperm forms after the polar nucleus is fertilized (pseudogamy). Thus, the apomictic seed maintains the same embryo:endosperm genomic ratio (2:3) as the sexual seed (Crane, 2001). Genetic analysis using segregating populations has led to the proposal of a simple genetic model for the inheritance of apomixis in this species in which apomixis is dominant over sexuality and is controlled by a single locus (Voigt and Bashaw, 1972; Voigt and Burson, 1992). In previous reports (Voigt and Bashaw, 1972; Voigt and Burson, 1992) the apomixis inheritance was evaluated only by progeny tests, having information of the complete process. At the moment the knowledge about the number of regions affecting apomixis in *Eragrostis* is limited due to the difficulties to evaluate parthenogenesis. The apomeiosis and parthenogenesis components of apomixis in other grasses are usually inherited together as a single dominant locus [apospory-specific genomic region (ASGR)] (Ozias-Akins and van Dijk, 2007), corresponding to a physically large hemizygous region of reduced recombination (Ozias-Akins et al., 1998; Stein et al., 2007). More recently, Conner et al. (2015) have found as a candidate gene for parthenogenesis in *Cenchrus/Pennisetum* an ASGR-BABY BOOM-like (ASGR-BBML). Recently, BBM was combined with other edited genes to produce an astounding progress in the transference of clonal reproduction to rice (Khanday et al., 2019; Wang et al., 2019) demonstrating the possibility of engineering key components of plant reproduction to allow the multiplication of heterotic combination through seeds.

For the past few years, we have been studying the molecular mechanisms involved in *E. curvula* reproduction, primarily using transcriptomic approaches. As a result, several differentially expressed genes between sexual and apomictic genotypes have been detected (Cervigni et al., 2008a,b; Selva et al., 2012, 2017; Garbus et al., 2017). Moreover, we demonstrated that the proportion of sexual embryo sacs in facultative apomicts increases under stress conditions, indicating that epigenetic and genetic mechanisms underlie the expression of apomixis in this species (Zappacosta et al., 2014; Rodrigo et al., 2017).

Genetic maps including molecular markers allow genetic regions linked to phenotypic characters to be identified and are therefore critical for genetic improvement (Collard et al., 2005). Single-nucleotide polymorphisms (SNPs) are currently the most widely used markers due to their abundance in the genome and the increasing ability to sequence large numbers of individuals in a cost-effective manner (Deschamps et al., 2012). Furthermore, in the past few years, genotyping-by-sequencing (GBS) has emerged as a new concept in marker development. The main advantages of GBS are its potential to detect SNP markers in numerous individuals and to combine genome reduction and barcode technology using a rapid, efficient, low-cost protocol for population mapping studies (Elshire et al., 2011). Worthington et al. (2016) developed the first saturated linkage maps for a polyploid apomict grass species (*Brachiaria decumbens*) using SNP markers generated by GBS. This saturated map has been used to assess synteny with foxtail millet and to identify flanking markers linked to the ASGR.

In this study, we constructed the first saturated linkage map at the tetraploid level for the diplosporous apomictic grass species *E. curvula* by combining traditional AFLP, SSR, and high-throughput molecular markers (GBS-SNP) and identified the localization of the diplospory controlling locus. We also used the saturated map to analyze the presence of regulatory regions affecting the expression of diplospory and the syntenic relationships with related grass species, shedding light on this agronomically important trait.

## Materials and Methods

### Plant Material

To obtain a segregating mapping population for the reproductive mode (F_1_ type progeny), the tetraploid sexual *E. curvula* genotype OTA-S (USDA accession PI574506; 2*n* = 4*x* = 40) was crossed with the facultative apomictic tetraploid cv Don Walter INTA. Plants of both parental genotypes were placed together in isolation in a confined sector of the greenhouse. We used one maternal plant (OTA-S) and three paternal clones (Don Walter). To ensure the cross-pollination, the pollen donor panicles were moved over the OTA-S panicles twice a day. Because it was impossible to perform castration (emasculation) due to the size and morphology of the spikes, some of the resulting seeds were produced by self-fertilization. The resulting seeds were sown in MS medium, and the germinated plants were transplanted to soil in pots and grown in the greenhouse. To confirm the hybrid origin of F_1_ individuals, fingerprinting analysis of RAPD male-specific amplicons was carried out as described by Rodrigo et al. (2017). Hybrid F_1_ plants were selected based on the presence of at least three paternal amplification bands using the primers shown in Supplementary Table S1. Selected individuals were cultivated in 10-L pots under greenhouse conditions with a photoperiod of 15 h light/9 h dark during the spring flowering period (Bahía Blanca, Buenos Aires Province, Argentina; 38° 42° S, 62° 16° W).

### Cytoembryological Analyses

To assess the reproductive modes of the F_1_ plants, megasporogenesis and megagametogenesis were analyzed according to Meier et al. (2011). Inflorescences were collected at the beginning of anthesis (when all embryo sac developmental stages are observable) and fixed in FAA (50% ethanol, 5% acetic acid, and 10% formaldehyde in distilled water). Individual spikelets were dehydrated in a tertiary butyl alcohol series and embedded in Paraplast (Leyca Paraplast Plus, United States). The samples were cut into 10-μm sections, stained with safranin-fast green, and observed under a Nikon Eclipse TE300 light transmission microscope (Tokyo, Japan). The reproductive mode was assessed by scoring the two main types of embryo sacs: octanucleated reduced *Polygonum*-type embryo sacs and tetranucleated nonreduced diplosporous *Eragrostis*-type embryo sacs. The latter contain an egg cell (2n), two synergids (2n), and one polar nucleus (2n) but lack antipodals (Meier et al., 2011). Plants were considered sexual when they showed only *Polygonum*-type embryo sacs and apomictic when at least one nonreduced diplosporous embryo sac was observed. At least 30 pistils with normally developed embryo sacs were analyzed per F_1_ individual.

### DNA Extraction

Genomic DNA was extracted from fresh leaf tissue according to Garbus et al. (2017). Briefly, fresh plant material was frozen and ground to a powder in liquid nitrogen using a TissueLyser II (Qiagen). For each sample, 100 mg of tissue was incubated at 65°C in preheated extraction buffer containing 100 mM Tris HCl pH 8, 1.4 M NaCl, 20 mM EDTA pH 8, 2% CTAB (w/v), and 0.5% (v/v) β-mercaptoethanol. Chloroform was subsequently added to reach a 2:1 ratio (buffer: chloroform), and the aqueous phase was collected after centrifugation. DNA was precipitated with one volume of isopropanol and washed with 70% (v/v) ethanol. The pellet was air-dried and resuspended in 50 μL of TE buffer containing 20 μg/ml RNase. DNA concentration was determined by spectrophotometry, and DNA quality was determined based on its integrity in agarose gels. All samples were quantified again using a Qubit Fluorometer (Thermo-Fisher Scientific) prior to library construction.

### SSR Markers

*E. curvula* SSR markers previously developed by Garbus et al. (2017) were used for genotyping of the mapping population; the primers are listed in Supplementary Table S1. PCR was performed in a final volume of 20 μl containing 1X Taq polymerase reaction buffer, 2.5 mM MgCl_2_, 0.125 mM of each dNTP, 1 μM of each primer, 50 ng of genomic DNA, and 2 U of Taq polymerase (Invitrogen, Brazil). The PCR program consisted of an initial denaturation at 94°C for 4 min, 35 cycles of 94°C for 30 s, 58°C for 1 min, and 72°C for 5 min, and a final extension at 72°C for 5 min. The PCR was performed in a thermocycler (MJ Research). Samples were mixed (2:1, v/v) with denaturing loading buffer (95% formamide and bromophenol blue), denatured at 95°C for 5 min, chilled on ice, and resolved in 6% (w/v) silver-stained polyacrylamide gels.

### AFLP Markers

AFLP markers were generated as described by Vos et al. (1995) with minor modifications. The sequences of the adapters and primers used for preamplification and selective amplification are shown in Supplementary Table S1. The amplification products were mixed with denaturing buffer, denatured at 95°C for 5 min, chilled on ice, and resolved in 6% (w/v) silver-stained polyacrylamide gels. The AFLP markers were given a number (primer combination) and a letter (indicating the order of polymorphic bands).

### GBS Library Preparation and Sequencing

A DNA GBS library was constructed for 86 F_1_ individuals (1 sample each), the two parental genotypes (4 samples each) and two controls. Genomic DNA (50 ng per individual) was processed as described by Elshire et al. (2011) at the Biotechnology Center (UWBC DNA Sequencing Facility, University of Wisconsin, Madison, United States). Digestion was carried out using the methylation-sensitive restriction enzyme *Ape*KI, followed by the ligation of barcoded adapters. The samples were pooled into one library that was PCR amplified. The library was sequenced to 100 bp in two lines of the Illumina HiSeq 2500 platform. Details can be found at the Biotechnology Center website^1^.

### GBS-SNP Discovery

The reads were trimmed using Cutadapt software version 1.14 (Martin, 2011) with the following parameters: (i) low-quality ends (−*q* = 20); (ii) maximum error rate (−*e* = 0.1); (iii) overlap length (−*O* = 1); and (iv) adapter (−*a* = AGATCGGAAGAGC). The trimmed reads were analyzed with FastQC software version 0.11.5 (Andrews, 2011). Using the barcodes provided by the sequencing service and the filtered reads, *de novo* SNP discovery and genotype calling were conducted using the UNEAK pipeline developed by Tassel software version 3.0 (Glaubitz et al., 2014) with the following parameters: (i) minimum number of reads (−*s* = 40,0000,000); (ii) enzyme used to create the GBS library (−*e* = *Ape*KI); (iii) minimum count of a tag to be output (−*c* = 10); (iv) error tolerance rate in the network filter (−*e* = 0.02); (v) minimum minor allele frequency (−mnMAF = 0.01); (vi) maximum minor allele frequency (−mxMAF = 0.5); (vii) minimum call rate (−mnC = 0.6); and (viii) maximum call rate (−mxC = 1). This method does not require a reference genome sequence because SNP discovery is performed directly within pairs of matched sequence tags and filtered through network analysis. To further avoid allele miscalling we considered the number of tags in each SNP for each individual, classifying an individual as homozygous when it has more than five tags and as heterozygous when it has at least one tag for each allele.

### Data Analysis and Linkage Map Construction

Segregation data from each parental genotype was analyzed independently. The configuration (homozygous/heterozygous) of all polymorphic markers (SSR, AFLP, and GBS-SNP) was recorded for each progeny. A *χ*^2^ test was used to determine the fit goodness (at *p* < 0.01) between the observed and expected number of genotypes. GBS-SNPs with unexpected alleles (e.g., one C/G SNP in one parent and C/C in the other showing G/G descendants) were excluded, even if they only had one offspring with the unexpected allele. Markers that were heterozygous in only one parent and had a segregation ratio of 1:1 (heterozygous:homozygous) in the progeny, were classified as single-dose allele (SDA) markers and used for map construction. Finally, GBS-SNP markers with more than 5% of missing data were removed.

There are several specific pipelines for polyploids such as polymapR (Bourke et al., 2018a) and TetraploidSNPMap (Hackett et al., 2017), but are designed to process markers with allelic dosage values, which are unavailable for GBS data. For this reason we decided to follow the traditional approach of a single dosage marker model (Li et al., 2014; Thaikua et al., 2016; Worthington et al., 2016). These markers possess a number of advantages over other marker segregation types, mainly in unexplored polyploid species for which the mode of inheritance is uncertain. Simplex markers allow an “assumption-free” linkage map to be created and the use of software designed for diploids (Bourke et al., 2018b). Thereby the genetic linkage maps were constructed for OTA-S and Don Walter using JoinMap 4.1 software (Van Ooijen, 2006) with the CP (cross-pollinator full-sib population) option. Markers with >98% identity were eliminated. Grouping analysis was carried out using a LOD (logarithm of odds) score threshold of 7.0 or higher. Maps were constructed within each linkage group using the regression-mapping algorithm, and map distance units were derived from the Kosambi mapping function with default options. Only linkages with a recombination frequency <0.40 were used for map construction.

### QTL Mapping

QTL mapping was performed using the Don Walter linkage map with MapQTL 6 software (Van Ooijen, 2009) using the Multiple QTL Mapping (MQM) method (Jansen, 1993, 1994; Jansen and Stam, 1994). Phenotypic data representing the proportion of diplosporous embryo sacs observed in each F_1_ hybrid (ranging from 0 in sexual individuals to 100 in apomictic individuals) were used. The LOD threshold to consider a QTL as significant was determined using permutation tests with 10,000 iterations and a genome-wide significance level of 0.05. MQM analysis was performed by setting a mapping step size of 1 cM and a LOD score higher than the threshold. Each significant QTL was characterized by its maximum LOD score, linkage group, position, percentage of explained phenotypic variation, and confidence interval extension (region at either side of the likelihood peak until the LOD score dropped to 2.0). The positions of QTLs on the genetic map were drawn using LinkageMapView software version 2.1.2 (Ouellette et al., 2017).

### Synteny Analysis

GBS-SNP markers sequences were queried against the genomes of *Oropetium thomaeum* (VanBuren et al., 2018), *Cenchrus americanus* (Varshney et al., 2017^2^), *Setaria italica* (Bennetzen et al., 2012^3^), *Zea mays* (Jiao et al., 2017), *Panicum hallii* (Bioproject: PRJNA250527), and *Oryza sativa (*Kawahara et al., 2013). Markers that aligned to the genomes with an identity >80% and a query coverage >70 were found using BLAST 2.7.1 (Altschul et al., 1990) and used to assign each linkage group to a chromosome and to identify homologs/homeologs groups. Given that the reference genomes used to establish syntenic relationships are available as haplotypes, the homologs/homeologs linkage groups are impossible to differentiate. Thereby we will mention them like “homologs/homeologs” since now. Circos v0.69 was used to plot synteny between the linkage maps and reference genomes (Krzywinski et al., 2009).

### Ploidy Level Analysis and Genome DNA Content Estimation

For ploidy level analysis, the parental plants, OTA-S and Don Walter, plus all the hybrid individuals were analyzed to corroborate its ploidy level. Cultivars Victoria and Don Eduardo, were used as diploid and hexaploid control, respectively. Approximately 0.5 cm^2^ of fresh leaf tissue was chopped with a sharp razor blade in extraction buffer (100 mM citric acid monohydrate and 0.5% [v/v] Tween 20). The suspensions were then filtered through nylon tissue with 42-μm mesh width. After filtration, samples were pooled in groups of four samples each. One ml of staining buffer (100 mM Tris–HCl, 5.3 mM MgCl^2^, 86 mM NaCl, 0.03 mM sodium citrate, 7.3 mM Triton X-100, 0.003 mM 4′-6-diamidino-2-phenylindole, pH 7.0) was added, and the tubes were stored in the dark on ice for 1 to 4 h before measurements. Fluorescence intensity of 4′-6-diamidino-2-phenylindole-stained nuclei was determined using the flow cytometer Ploidy Analyser PA (Partec, Germany).

For genome DNA content estimation, approximately 0.5 cm^2^ of fresh *E. curvula* leaf tissue, together with an equal amount of *Secale cereale* cv. Dankovske leaf tissue, was chopped with a sharp razor blade in extraction buffer (5 mM Tris, 2 mM Na_2_EDTA, 80 mM KCl, 20 mM NaCl, 15 mM β-mercaptoethanol, and 0.1% [v/v] Triton X-100, pH 7.5). The nucleus suspension was filtered and incubated in 100 μl of staining solution consisting of 100 mg/l propidium iodide (PI) stain and RNase A. The stained nucleus suspension was analyzed using a flow cytometer (Partec, Germany). Genome size was estimated based on the corresponding mean value for *S. cereale* cv. Dankovske (16.19 pg 2C DNA content, Doležel et al., 1998).

## Results

### Mapping Population Development and F_1_ Phenotyping

A total of 300 offspring derived from the cross between OTA-S and Don Walter-INTA were obtained. In the first hybrid selection using RAPD markers, 86 plants were selected (Supplementary Figure S1), but 19 were ultimately eliminated because they originated from self-pollination of the female plant by the mentor effect of the male pollinator. Five other individuals were eliminated due to a failure in GBS genotyping (low read counts, see below). Consequently, the mapping population consisted of 62 hybrids. The tetraploid level of the parental and hybrid plants was corroborated by flow cytometry.

**FIGURE 1:**
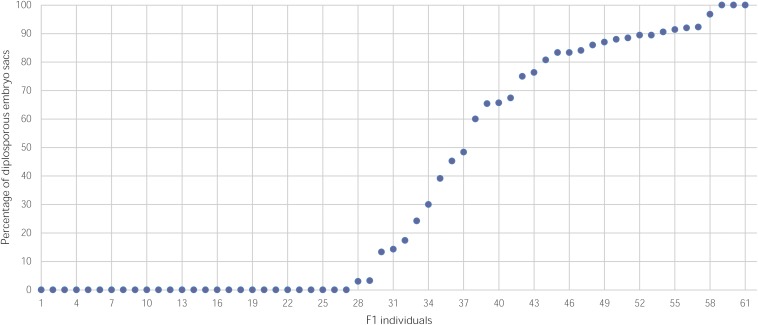
Percentage of diplosporous embryo sacs in individuals of a mapping population derived from the OTA-S x Don Walter INTA cross.

Cytoembryological analysis (Supplementary Figure S2) of 61 individuals of the population (one hybrid did not flower) gave a ratio of apomictic versus sexual individuals of 1:1 (34:27, *X*^2^ = 0.37), which agrees with the model of inheritance of a single dominant genetic factor. Figure 1 shows the distribution of hybrid plants according to their reproductive mode (2,850 sexual and apomictic pistils observed). Interestingly, the proportion of sexual embryo sacs within the apomictic plants varied from 0 to 97%, indicating that apomixis in *E. curvula* is a characteristic with highly variable expressivity.

### GBS-SNP Identification

The sequencing of the library produced 366,193,356 (100 bp) reads (Bioproject: PRJNA509552). After trimming, 33,350,780 low-quality reads were removed, and 332,842,576 reads were subsequently analyzed using the UNEAK pipeline. Five samples (Z116, Z154, Z217, Z223, and Z252) were eliminated from further analyses due to the low number of reads. The depth of coverage for each sample is listed in Supplementary Table S2.

A total of 332.8 million of reads were assigned to 178,559 tag pair sites. After removing the markers with missing data in the parental plants, 106,105 GBS-SNP markers were identified. Segregating GBS-SNP markers that were heterozygous in one parent and homozygous in the other one were selected, resulting in 28,074 and 33,765 GBS-SNPs for OTA-S and Don Walter, respectively. Table 1 shows the results obtained after sequential filtering of these data. The final number of GBS-SNP markers was 1,447 for OTA-S and 2,192 for Don Walter (Table 1). After including 11 SSR and 93 AFLP markers (as shown in Table 2), the final number of markers was 1,489 for OTA-S and 2,255 for Don Walter (including the phenotype in the last case).

**TABLE 1:** Steps in the GBS-SNP marker filtering procedure and the number of markers selected in each step for each parental plant (OTA-S and Don Walter) of the *E. curvula* mapping population.

**GBS-SNP marker**	**OTA-S**	**Don Walter**
Heterozygous for each parental plant	28,074	33,765
Without unexpected alleles	22,648	28,425
Single dose alleles (1:1 segregation)	9,829	11,991
Missing data ≤5%	1,447	2,192

**TABLE 2:** Final number of markers for each parental plant (OTA-S × Don Walter) of the *E. curvula* mapping population.

**Marker**	**OTA-S**	**Don Walter**
GBS-SNP	1,447	2,192
SSR	6	5
AFLP	36	57
Diplospory		1
Total	1,489	2,255

### Genetic Linkage Map Construction

We constructed two linkage maps corresponding to the female (OTA-S) and male (Don Walter) parent using JoinMap 4.1. As a first step, identical markers were excluded (54 and 78 identical markers were eliminated from the OTA-S and Don Walter data, respectively). The high level of heterozygosity and the maximum number of markers per linkage group allowed by the regression method in the JoinMap software resulted in more groups than the expected ones (20). Thereby, we used the 2n chromosome number to define the linkage group number as other authors previously did (Li et al., 2014; Worthington et al., 2016).

**FIGURE 2:**
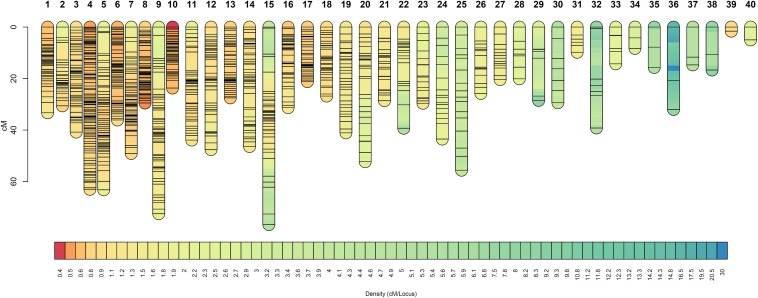
Linkage groups of the sexual plant OTA-S (*E. curvula*) obtained using GBS-SNPs, SSRs, and AFLPs. Marker positions are expressed in centimorgans. Different colors represent different marker densities.

**FIGURE 3:**
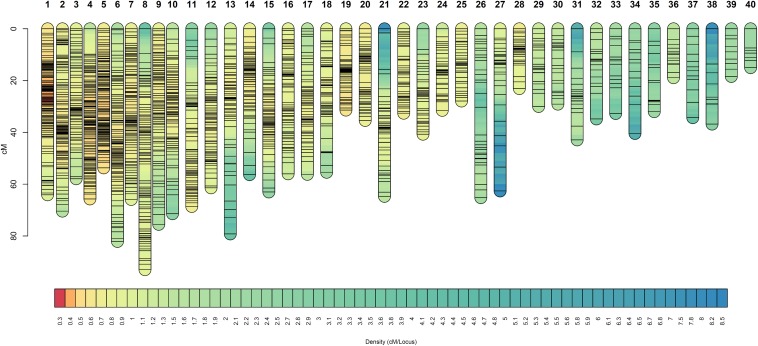
Linkage groups of the facultative apomictic plant Don Walter (*E. curvula*) obtained using GBS-SNPs, SSRs, and AFLPs. Marker positions are expressed in centimorgans. Different colors represent different marker densities.

The OTA-S map was defined by 1,114 SDA markers distributed in 40 linkage groups (LOD score threshold 7.0 or 9.0, Supplementary Table S3), which is consistent with the number of chromosomes, and contained a minimum of 2 and a maximum of 102 markers per linkage group (Table 3, Figure 2, and Supplementary Figure S3). The total length of the OTA-S map was 1,335 cM, with an average marker density of 1.22 cM per marker. The genetic linkage map of the apomictic parent Don Walter was built using 2,019 SDA markers distributed in 40 linkage groups (LOD score threshold 7.0 or 8.5, Supplementary Table S3), with 7–123 markers per linkage group (Table 3, Figure 3, and Supplementary Figure S4). The total length of the Don Walter map was 1,976.2 cM, with an average of 0.98 cM per marker. In total, more than 90% of the interlocus gaps in both genetic maps were <4 cM, and only seven and four gaps were >10 cM in the OTA-S and Don Walter linkage maps, respectively. The order and exact positions of markers on the maps are shown in Supplementary Figures S3, S4, and the GBS-SNPs sequences in Supplementary Table S4.

**TABLE 3:** Distribution of single-dose allele (SDA) markers across the 40 linkage groups on the *E. curvula* (OTA-S and Don Walter) genetic maps.

**LG**	**OTA-S**		**Don Walter**	
		
	**No. of markers**	**Distance (cM)**	**No. of markers**	**Distance (cM)**
1	43	33.3	123	64.1
2	28	30.6	103	70.4
3	47	40.8	66	57.9
4	101	63.0	118	65.7
5	65	63.2	109	53.7
6	62	36.1	103	82.1
7	57	49.1	97	65.9
8	54	29.5	94	93.0
9	53	72.5	84	75.5
10	45	23.7	75	71.4
11	46	43.9	73	68.6
12	45	47.6	65	61.4
13	42	27.5	63	79.2
14	42	46.4	62	56.3
15	41	76.7	62	63.0
16	37	31.3	61	56.0
17	32	21.2	58	56.2
18	31	26.8	54	55.4
19	30	41.1	51	31.4
20	29	52.3	50	35.4
21	24	28.6	46	64.7
22	19	39.3	45	32.6
23	18	29.6	36	40.7
24	17	43.5	35	31.5
25	16	55.6	33	27.9
26	15	25.8	31	65.2
27	13	20.4	28	62.6
28	8	20.1	28	23.1
29	8	28.5	21	30.0
30	7	29.4	20	29.1
31	7	9.8	19	42.9
32	7	39.2	17	34.8
33	6	14.3	13	32.7
34	3	8.3	13	40.4
35	3	15.7	13	32.0
36	3	32.0	13	18.9
37	3	14.7	12	34.3
38	3	16.7	9	36.8
39	2	1.6	9	18.4
40	2	5.0	7	15.0
Total	1,114	1,335.0	2,019	1,976.2

**FIGURE 4:**
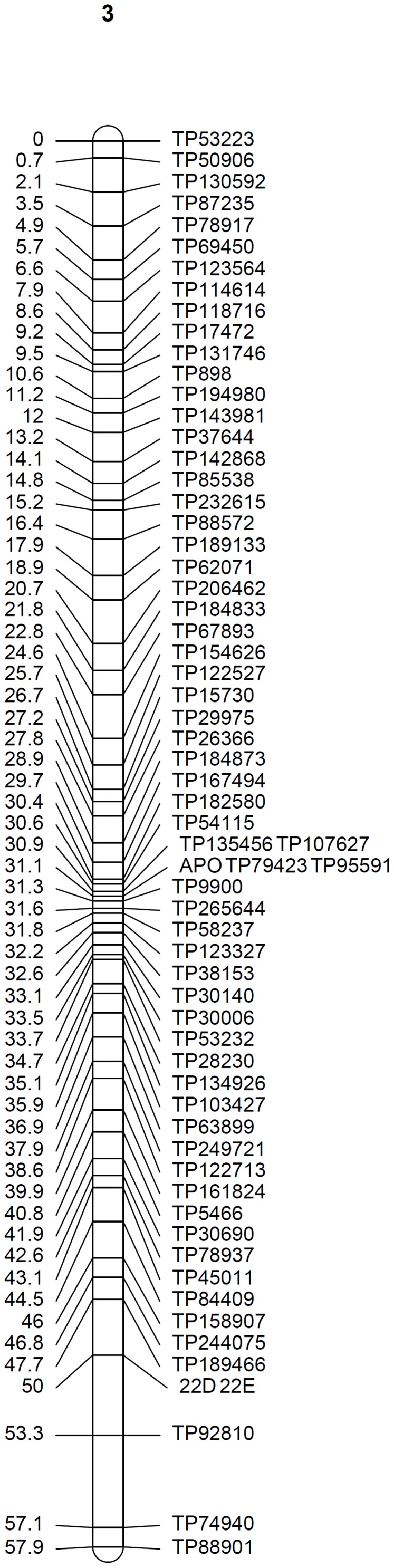
Linkage group 3 from the facultative apomictic cv. Don Walter (*E. curvula*) containing the locus that controls diplospory (APO). Marker positions are expressed in centimorgans.

At the mapping threshold stated, the diplospory locus was mapped to Don Walter linkage group 3, along with other 65 markers (Figure 4). This locus was flanked by four GBS-SNPs having a recombination frequency of zero, being in agreement with previous reports of a low recombination region controlling the trait in other species (Ozias-Akins and van Dijk, 2007; Albertini et al., 2010; Ortiz et al., 2013).

### QTL Analysis to Identify Regions Affecting Diplospory Expressivity

**FIGURE 5:**
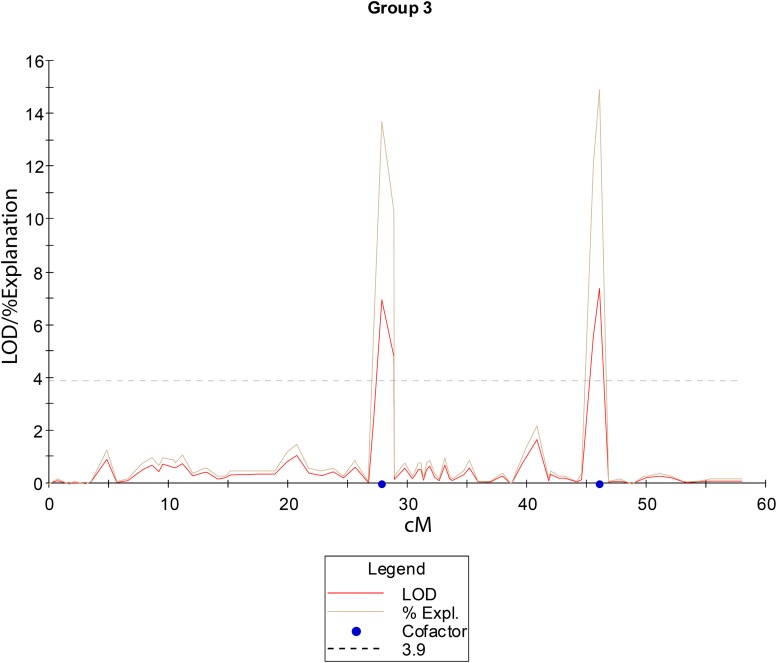
Linkage group 3 from the facultative apomictic cv. Don Walter (*E. curvula*) showing the QTLs positions (in cM) for diplospory. The LOD threshold to consider a QTL as significant is indicated as a dashed line at a LOD value of 3.9.

To detect loci associated with the expressivity of diplospory in *E. curvula*, we performed interval-mapping analysis using the phenotypic information derived from cytoembryological analysis of F_1_ hybrids (see above) and the genetic linkage map of Don Walter. This analysis detected two genomic regions highly associated with this trait (LOD score >3.9) in Don Walter linkage group 3 (Figure 5 and Supplementary Figure S5). The maximum LOD scores for each potential QTL were 6.96 to 7.39, explaining an estimated phenotypic variation (R^2^) of 13.7 and 14.9%, respectively (Table 4). One of these two regions is very close to the diplospory locus that was mapped using JoinMap (located at 3.27 cM); thus, it could be considered the major determinant of this trait. The second region is located 15 cM from the diplospory locus. Three additional QTLs were found with a LOD >3 but lower than the threshold value (LOD >3.9). Two of these QTLs were localized to linkage group 1 and the other to linkage group 20 (Table 4 and Supplementary Figure S5). The positions and quantitative information about these QTLs are shown in Table 4.

**TABLE 4:** QTLs mapping for diplospory on the facultative apomictic cv. Don Walter (*E. curvula*) linkage groups, showing only the QTL with LOD values higher than 3.

**Marker**	**Max LOD**	**Linkage group**	**Position (cM)**	**Phenotypic variation (%)**	**Confidence Interval** (cM)
TP10944	3.01	1	1.00	3.8	2.41
TP157277	3.2	1	8.90	4.0	2.20
TP26366	6.96	3	27.84	13.7	1.67
TP158907	7.39	3	46.03	14.9	2.27
TP89963	3.02	20	31.63	3.8	7.38

*The LOD threshold to consider a QTL as significant was established at a LOD value of 3.9.*

### Syntenic Analysis to Identify Homolog/Homeolog Groups

**FIGURE 6:**
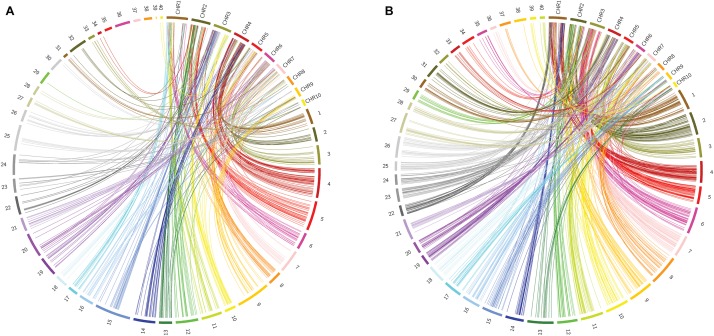
Circos graphs comparing the *E. curvula* linkage maps with the *O. thomaeum* physical map (**A:** OTA-S, **B:** Don Walter). The numbers from 1 to 40 represent the linkage groups of the sexual and apomictic *E. curvula* plants and the labels CHR1-10 represent the chromosomes of *O. thomaeum*.

**FIGURE 7:**
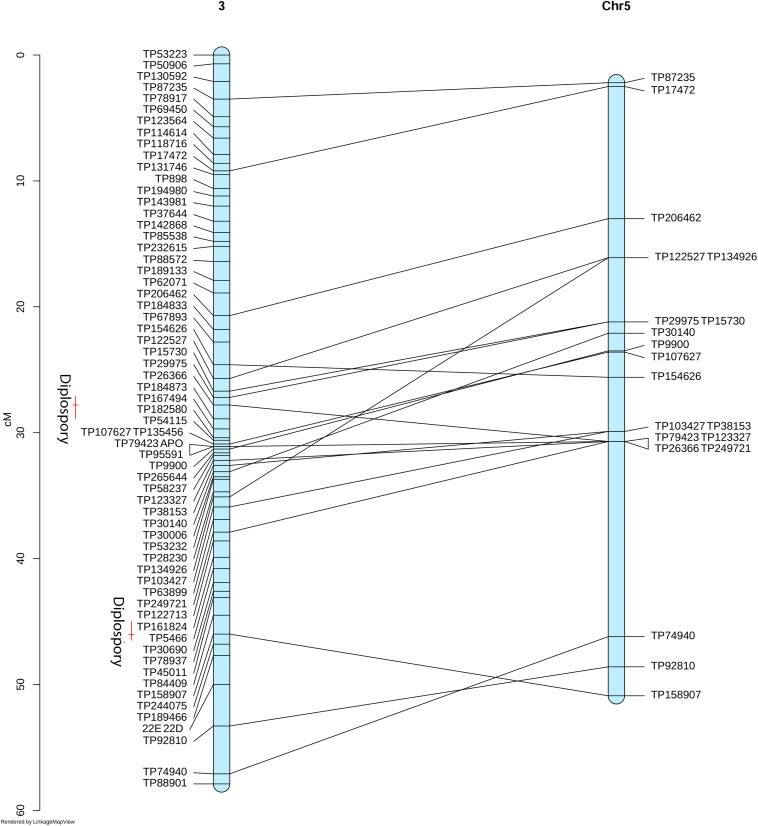
Synteny between the *E. curvula* (facultative apomictic cv. Don Walter) linkage group 3 with the *O. thomaeum* chromosome 5. Red bars represent the QTLs positions for diplospory.

To identify homologs/homeologs groups in the linkage maps, we mapped the sequences of the GBS-SNP markers against the genomes of other species. Analysis using *Oropetium thomaeum* as a reference (the closest species with a high-quality genome sequence; VanBuren et al., 2018) showed that 477 (40%) and 900 (45%) GBS-SNP markers from OTA-S and Don Walter, respectively, mapped to unique positions (best match) under the above-mentioned conditions (identity >80% and query coverage >70). Although the order of the markers and their positions in the *O. thomaeum* genome are not highly correlated with those of *E. curvula*, analysis of Circos graphs showed that the markers of each linkage group tended to cluster on the same chromosome (Figure 6). As an example we can mention OTA-S linkage group 4 that matches mainly with *O. thomaeum* chromosome 4 (dark red lines in Figure 6A). From the male side (cv. Don Walter), linkage group 5 of *E. curvula* matches with *O. thomaeum* chromosome 3 (red lines in Figure 6B). Nonetheless, it is possible to observe groups that match with more than one chromosome, like Don Walter linkage group 8 matches with *O. thomaeum* chromosomes 7 and 8. On the other hand, as is shown in Figure 7, markers of Don Walter linkage group 3 (containing the diplospory locus) are syntenic with those of *O. thomaeum* chromosome 5. Most of the linkage groups showed homology, primarily with a single chromosome of *O. thomaeum* (Supplementary Tables S5, S6). Table 5 shows the groups of homologs/homeologs considered to be exclusive linkage groups (in which most common markers fell into a single chromosome) or shared linkage groups (in which most markers were divided into two or three chromosomes). This enabled us to identify homologs/homeologs groups for each linkage map, to establish the relationship between the two maps, and to validate the genetic *E. curvula*-saturated maps.

**TABLE 5:** Homologs/homeologs linkage groups from *E. curvula* obtained by synteny analysis with the *O. thomaeum* physical map.

***O. thomaeum* chromosome**	**OTA-S**		**Don Walter**	
		
	**Exclusive LG**	**Shared LG**	**Exclusive LG**	**Shared LG**
1	6, 11, 17	16, 32	1, 4, 14, 22	
2	5, 12, 13, 34	16, 24	2, 9, 12, 36	13, 34, 35
3	10, 15, 18, 19, 22, 28	16, 32	5, 6, 25, 29	34, 35
4	1, 2, 4, 14, 40	24	7, 19, 23, 31	24, 40
5	8, 26, 29	32	3, 11, 26, 30	
6	3, 20		16, 20, 28, 33	
7	7, 21, 31		10, 18, 32, 38	8
8	24, 25	16	21, 37	8
9	9, 23, 30		15, 17, 27	40
10	27, 33	24	39	13, 24, 35

*Exclusive LG indicate the groups where most of the markers match to a single chromosome. Shared LG indicate the groups where most of the markers match with multiple chromosomes.*

We also analyzed synteny with genomes of other related species (*C. americanus*, *O. sativa*, *P. hallii*, *S. italica*, and *Z. mays*), yielding similar results to those described above (Supplementary Tables S7, S8). When we compared the markers completely linked to the *E. curvula* apolocus (TP135456, TP107627, TP79423, and TP95591) with the genome of related species, we found that the GBS-SNP sequences gave homology with *O. thomaeum* (Chr5), *C. americanus* (Chr4), *O. sativa* (Chr5), *P. hallii* (Chr5), *S. italica* (Chr3), and *Z. mays* (Chr6 and Chr8) (Supplementary Table S9).

Finally, we evaluated the genomic DNA content of the parents of the mapping population. Flow cytometry analysis yielded an estimated haploid genome size of 1,312 Mbp for OTA-S and 1,195 Mbp for Don Walter.

## Discussion

Segregation analysis of the reproductive mode in our *E. curvula* tetraploid mapping population revealed a 1:1 ratio of apomictic versus sexual individuals. This type of inheritance supports the hypothesis that diplospory is controlled by a single dominant genetic factor in *E. curvula*, as described for other diplosporous apomictic species, such as *Taraxacum officinale* (Vijverberg et al., 2004) and *Tripsacum dactyloides* (Grimanelli et al., 1998). Pioneering studies of the inheritance of apomixis in weeping lovegrass were carried out by Voigt and colleagues (Voigt and Bashaw, 1972; Voigt and Burson, 1992), who phenotyped plants by measuring various morphological traits, obtaining a ratio of apomictic versus sexual offspring of 1:1.4. The authors proposed a simple genetic model for the inheritance of apomixis in weeping lovegrass, i.e., apomixis is dominant over sexuality and is controlled by a single gene. Voigt et al. (Voigt and Bashaw, 1972; Voigt and Burson, 1992) categorized plants into apomictic, highly sexual, and sexual, but Savidan (2000) later proposed that plants are apomictic even if they only have the ability to produce apomictic offspring. When we reanalyzed Voigt et al.’s results taking into account the concept proposed by Savidan (2000), the proportion changed to 1.7:1 (96:56). The results obtained in this study using cytoembryological observations and molecular markers (both methods are more reliable than the analysis of morphological traits) showed that a single locus controls diplospory in weeping lovegrass and that this trait is dominant over sexuality. Whether the expression of this trait relies on a single gene or linked cosegregating genes is still unknown.

In other diplosporous species, although the regions controlling different components of apomixis (apomeiosis, parthenogenesis, and autonomous or pseudogamous endosperm) are physically separated, these regions are inherited either as a single locus (*T. dactyloides*) or independently (*E. annuus*) (Grimanelli et al., 1998; Noyes et al., 2007). In the case of apospory, a more frequent apomixis mechanism than diplospory, a dominant locus of simple inheritance has been identified (Akiyama et al., 2004; Calderini et al., 2006, Okada et al., 2011; Ortiz et al., 2013), although in *Poa pratensis*, two genetic factors are thought to control apospory and parthenogenesis (Albertini et al., 2001). In *Pennisetum squamulatum* and in species from the genus *Paspalum*, the ASGR shows a lack of recombination, forming an extensive block (50 Mbp in *P. squamulatum*; Akiyama et al., 2004) that is fully inherited, thus ensuring the concurrent inheritance of all its components (Ozias-Akins et al., 1998; Labombarda et al., 2002; Stein et al., 2007). Several authors have reported the presence of repetitive elements, pseudogenes, and heterochromatic regions in the ASGR. Koltunow and Grossniklaus (2003) hypothesized that the repetitive sequences act as a sink to sequester factors involved in the sexual reproductive pathway, thereby altering the expression of sexual reproductive processes and possibly causing apomixis. More recently, Kotani et al. (2013) reported that extensive repetitive sequence structures associated with the apospory locus in *Hieracium* are not required for apomixis. Therefore, it is possible that these structural features and allele divergence occur as a consequence of asexual reproduction and suppressed recombination, which might have evolved to maintain the genetic elements required for apomixis.

Although several reports describe the presence of genes in diverse apomictic species, which are differentially expressed or play functional roles in apomictic development (Albertini et al., 2005; Corral et al., 2013; Siena et al., 2014; Conner et al., 2015; Pellegrini, 2016; Worthington et al., 2016; Garbus et al., 2017; Selva et al., 2017), little is known about the gene or genes that control regulatory programs or common pathways among different apomictic species or that trigger the mechanisms underlying apomixis.

In this study, we constructed genetic linkage maps for *E. curvula*, including one for the female sexual parent and one for the male apomictic parent. These maps are the most saturated maps for the genus *Eragrostis* and some of the most saturated maps for polyploid forage grass and apomictic species produced to date (Jessup et al., 2003; Stein et al., 2007; Thaikua et al., 2016; Worthington et al., 2016). Nonetheless, additional studies are needed to allow our linkage maps to reach the high resolution of genetic maps of model species, which include thousands of markers mapped with high accuracy and precision. One of the greatest limitations to the construction of the linkage genetic maps produced in this study was the small number of individuals in the mapping population, i.e., 62. A population size >75 should be used (Wu et al., 1992), but this was difficult to achieve for *E. curvula* due to a variety of factors, such as the complex reproductive mode of this species, the inability to perform castration (emasculation) due to spike size and morphology, and the high frequency of self-pollination in the single tetraploid sexual genotype available (OTA-S). In addition, many other genotypes used as pollen donors were incompatible with the maternal plant. Despite these limitations, this is a high density map which is consistent with data collected by other authors using similar models and techniques (Worthington et al., 2016, Huang et al., 2018).

Several linkage maps of polyploid species are based exclusively on markers that segregate at a 1:1 ratio (SDA), enabling the use of diploid mapping softwares like JoinMap. Allopolyploid species have disomic inheritance, and its genetics is therefore similar to that of diploids, except for the presence of multiple genomes. The assumption that all the markers have a 1:1 disomic inheritance might be an oversimplification because the markers with a different segregation pattern were not considered. However, for our dataset, this is a straightforward approach very well documented in the literature to deal with GBS-SNP markers in allotetraploid species (Li et al., 2014; Thaikua et al., 2016; Worthington et al., 2016).

*E. curvula* is considered to be an allopolyploid species, although multivalent formation has also been recorded in some polyploid genotypes, such as Tanganyika and Don Eduardo (Vorster and Liebenberg, 1977; Poverene, 1988). However, multivalents are not frequent in the parental genotypes of the mapping population, where preferential pairing among primary homologs has been demonstrated (Poverene, 1988).

The sizes of the linkage maps of OTA-S and Don Walter are quite different (1,335 cM versus 1,976.2 cM, respectively). This variation in genetic map size is not related to the difference in genome size between genotypes, as we estimated the haploid genome sizes to be 1,312 Mbp for OTA-S and 1,195 Mbp for Don Walter. Thus, although the variation in the genetic sizes of the linkage group maps of both parents is not likely due to differences in genome size, this variation might reflect the differential recombination rates of the genotypes. Indeed, studies of model plants have demonstrated the impact of genome sequence divergence on recombination rates, with a lower recombination rate related to higher levels of genome divergence (Chetelat et al., 2000; Opperman et al., 2004; Li et al., 2006). In addition, recombination rates are known to differ between sexes in both plants and animals (Lorch, 2005). For example, Huang et al. (2018) found that the male genetic map of Clementine mandarin was notably larger than its female counterpart. Another possible reason for the difference in the sizes of the linkage maps is that OTA-S was obtained by bulk seed harvest produced in isolation from four tetraploid (2*n* = 40) clones derived from PI 299929 and from a cross between PI 299928 and PI 299929 (Voigt, 1976).

When we investigated synteny of the *E. curvula* genome with genomes of other related species, we identified homologs/homeologs linkage groups when the OTA-S and Don Walter linkage maps were compared with the physical map of *O. thomaeum*, the closest relative with a high-quality genome sequence. Using this information, it was not only possible to obtain homologs/homeologs groups for each map but also to establish which groups of each map would be equivalent. The synteny analysis of the apolocus linked markers with the genome of related species showed an interesting result. The maize relative diplosporous genus *Tripsacum* have two RFLP markers (*csu*68 and *umc*28) linked to diplospory that are located at a distal position on *Z. mays* Chr6L (Leblanc et al., 1995). This region is syntenic to *Z. mays* Chr8 and Chr3 (Savidan et al., 2004). Thereby, our results are promising since the *E. curvula* apolocus linked markers gave homology with regions located on maize Chr6L and Chr8. Regarding to other species, our markers gave homology with chromosomes or genomic regions that are different to the ones reported in the literature as linked to the apolocus (see Table 6). These findings supports the hypothesis that apomixis is polyphyletic and emerged several times during evolution (Carman, 1997).

**TABLE 6:** Synteny between the apolocus region reported in apomictic species and non-apomictic reference species.

**Species**	**Apomixis mechanism**	**Synteny**	**References**
*Brachiaria decumbens*	Apospory	*Setaria italica* (Chr5)	Worthington et al., 2016
*Brachiaria humidicola*	Apospory	*Setaria italica* (Chr1)	Worthington et al., 2019
*Brachiaria brizantha*	Apospory	*Oryza sativa* (Chr2)	Pessino et al., 1998
		*Zea mays* (Chr5)	Pessino et al., 1997
		*Setaria italica* (Chr1)	Zhang et al., 2012
*Pennisetum squamulatum*	Apospory	*Setaria italica* (Chr2)	Sapkota et al., 2016
*Paspalum simplex*	Apospory	*Oryza sativa* (Chr12)	Pupilli et al., 2001
		*Setaria italica* (Chr3)	Galla et al., 2019
*Paspalum malacophyllum*	Apospory	*Oryza sativa* (Chr12)	Pupilli et al., 2004
*Paspalum notatum*	Apospory	*Oryza sativa* (Chr2, Chr12)	Pupilli et al., 2004
*Paspalum procurrens*	Apospory	*Oryza sativa* (Chr12)	Hojsgaard et al., 2011
*Tripsacum*	Diplospory	*Zea mays* (Chr6)	Leblanc et al., 1995
*Eragrostis curvula*	Diplospory	*Oryza sativa* (Chr5)	This study
		*Setaria italica* (Chr3)	
		*Zea mays* (Chr6, Chr8)	

*For *E. curvula* the syntenic analysis was done with the markers cosegregating with the apolocus.*

The apomictic plants in our mapping population showed different levels of expression of diplospory, with 3–100% of the observed pistils having apomictic embryo sacs. We previously reported (Rodrigo et al., 2017) that OTA-S only shows *Polygonum*-type embryo sacs, whereas Don Walter is a facultative genotype, with 60–100% diplosporous apomictic embryo sacs. As occurs in most known apomictic plants, these plants are facultative and can switch their developmental program back and forth from the asexual to the sexual route (Brukhin, 2017). This trait appears to be useful for the evaluation of candidate genes, especially genes with quantitative effects. Other studies, such as the one of Noyes (2005) related with the inheritance of diplospory in *Erigeron*, have also found a complete gradient of apomixis expression. These different levels of expression of diplospory observed in *E. curvula* allowed us to evaluate diplospory as a quantitative trait and to look for other genomic regions that could regulate it. Our QTL analysis revealed two main regions very close to the diplospory locus in Don Walter linkage map (linkage group 3) and three other regions with a LOD value slightly below the LOD significance threshold, including two localized in linkage group 1 and the other in linkage group 20. Although the phenotypic analyses were performed in only one environment, the results are trustable because are in concordance with the linkage mapping analysis and the diplospory locus position. Additionally, to the best of our knowledge this study is the first conducted to date that treats apomixis as a quantitative trait and provides evidence for an external region that regulates this trait. Another important finding in favor of the presence of regions that regulate this trait is that sexual/apomixis expressiveness is strongly dependent on environmental conditions (Zappacosta et al., 2014; Rodrigo et al., 2017), which, in turn, could be indicative of regulation at the epigenetic level.

*Eragrostis*-type apomixis has particular characteristics that make it an interesting model for the transfer of apomixis, especially for crops such as maize, which are highly sensitive to changes in the embryo:endosperm ploidy ratio, which should be equal to 2:3. In our model, this ratio is the same as that of sexual endosperm. This is an important difference from other apomictic models in which the situation is variable and relaxed (the endosperm can develop under a wide range of relationships) (Hojsgaard, 2018). The embryo:endosperm ploidy ratio is strictly 2:3 in several model species because at the early stages of embryo and endosperm development, many alleles are silenced (imprinted) depending on their parental origin. Any deviation in the dosage will result in the arrest of endosperm development and seed abortion (Brukhin, 2017). Other interesting aspect of this model is that the *Eragrostis* type embryo sac development lacks of meiotic stages (Crane, 2001), and as a diplosporous plant, compared to apospory, the chances of polyembryony are even lower (Asker and Jerling, 1992; Koltunow and Grossniklaus, 2003; Batygina and Vinogradova, 2007).

## Conclusion

Phenotyping of an F_1_ population showed that the segregation of diplospory follows a 1:1 (apomictic:sexual) ratio, indicating that a single gene or genomic region is involved in diplospory.

We constructed the first genetic map of *E. curvula*. This map is the most saturated map for the genus *Eragrostis* and one of the most saturated maps for a polyploid forage grass and apomictic species constructed to date, with 40 linkage groups per parent. These results are somewhat expected for an allotetraploid with a high grade of heterozygosis.

Our linkage analysis determined that the diplospory locus and other 65 markers in a single linkage group (Don Walter LG3). This locus is closely flanked by two QTLs that could be linked to the expressivity of this trait.

The use of the current mapping population gave us the opportunity to construct a genetic map and to locate molecular markers associated with apomixis. Furthermore, this population is composed of individuals that are genetically close but have different reproductive modes, which might allow us to conduct further expression studies that will help identify candidate genes that regulate apomixis. This also should allow us to map other traits that are contrasting in the parental genotypes and are limiting factors for weeping lovegrass production, such as forage quality, a trait related to lignin content. Finally, it might also be possible to map genes involved in biotic and abiotic stress tolerance; these are critical traits in the breeding of this forage grass, which is cultivated in marginal crop regions.

Further studies using the auxin test proposed by Matzk (1991) to evaluate parthenogenesis will allow us to determine if both traits - diplospory and parthenogenesis - are controlled by genes located in one or more genomic region/s.

## Author Contributions

DZ, MM, and VE conceived and designed the study. DZ, JR, and JPS developed the mapping population. DZ, MM, JR, and JO phenotyped mapping population. JC trimmed GBS data and made GBS-SNP discovery. DZ, JG, MM, JS, and JO performed genetic mapping. DZ, JG, and CG performed synteny and QTLs analysis. All authors participated in manuscript elaboration. VE and EA conducted and supervissed the research, obtained funding and participated in manuscript writing.

## Conflict of Interest Statement

The authors declare that the research was conducted in the absence of any commercial or financial relationships that could be construed as a potential conflict of interest. The handling Editor is currently organizing a Research Topic with one of the authors EA and confirms the absence of any other collaboration.

## Footnotes

1 https://www.biotech.wisc.edu/services/dnaseq

2 https://www.ncbi.nlm.nih.gov/bioproject/PRJNA294988

3 https://www.ncbi.nlm.nih.gov/bioproject/PRJNA32913

## Supplementary Material

FIGURE S1 Selection of hybrid *E. curvula* plants with RAPD markers. Fragments were amplified with primer 248 and revealed in 6% acrylamide gels. Arrows indicate polymorphisms between the parental plants and the stars show offsprings harboring paternal markers.

FIGURE S2 Development of the sexual **(A–C)** and diplosporic embryo sacs **(D–F)** in plants of weeping lovegrass. Bar: 50 μm. Sections dyed with safranina-fast green. **(A)** Megaspore mother cell and degenerated megaspores, **(B)** Binucleated embryo sac, **(C)** Tetranucleate embryo sac, **(D)** Elongated megaspore mother cell, **(E)** Binucleate embryo sac, **(F)** Tetranucleate embryo sac.

FIGURE S3 Linkage groups of the sexual plant OTA-S (*E. curvula*) obtained using GBS-SNPs, SSRs, and AFLPs. Marker positions are expressed in centimorgans.

FIGURE S4 Linkage groups of the facultative apomictic plant Don Walter (*E. curvula*) obtained using GBS-SNPs, SSRs, and AFLPs. Marker positions are expressed in centimorgans.

FIGURE S5 Linkage groups from the facultative apomictic cv. Don Walter (*E. curvula*) showing the QTLs positions (in cM) for diplospory. The LOD threshold to consider a QTL as significant is indicated as a dashed line at a LOD value of 3.9.

TABLE S1 RAPD and SSR primers and AFLP adaptors, pre-amplification and selective primer sequences used to construct the *E. curvula* genetic maps.

TABLE S2 Number of reads, length and coverage (average genome size of 1,250 Mb) of the GBS library from *E. curvula* used for SNP calling. Barcodes for each sample are indicated.

TABLE S3 LOD score where each linkage group was determined.

TABLE S4 SNPs names and the alternative sequences of each alleles.

TABLE S5 Synteny between the *E. curvula* sexual genotype OTA-S linkage groups and the physical map of *O. thomaeum*.

TABLE S6 Synteny between the *E. curvula* facultative apomictic cultivar Don Walter linkage groups and the physical map of *O. thomaeum*.

TABLE S7 Synteny between the *E. curvula* sexual genotype OTA-S linkage groups and the physical maps of *Cenchrus americanus*, *Oryza sativa*, *Panicum hallii*, *Setaria italica*, and *Zea mays*.

TABLE S8 Synteny between the *E. curvula* facultative apomictic cultivar Don Walter linkage groups and the physical maps of *C. americanus*, *O. sativa*, *P. hallii*, *S. italica*, and *Z. mays.*

TABLE S9 Homology of GBS-SNP markers of *E. curvula* facultative apomictic cultivar Don Walter with the chromosomes of *Oropetium thomaeum*, *C. americanus*, *O. sativa*, *P. hallii*, *S. italica*, and *Z. mays*.
