# Supplementary figures and images for: A High-Density Linkage Map of the Forage Grass Eragrostis curvula and Localization of the Diplospory Locus
Source: Front Plant Sci. 2019 Jul 12;10:918. doi: 10.3389/fpls.2019.00918 (PMC6640543; doi:10.3389/fpls.2019.00918)

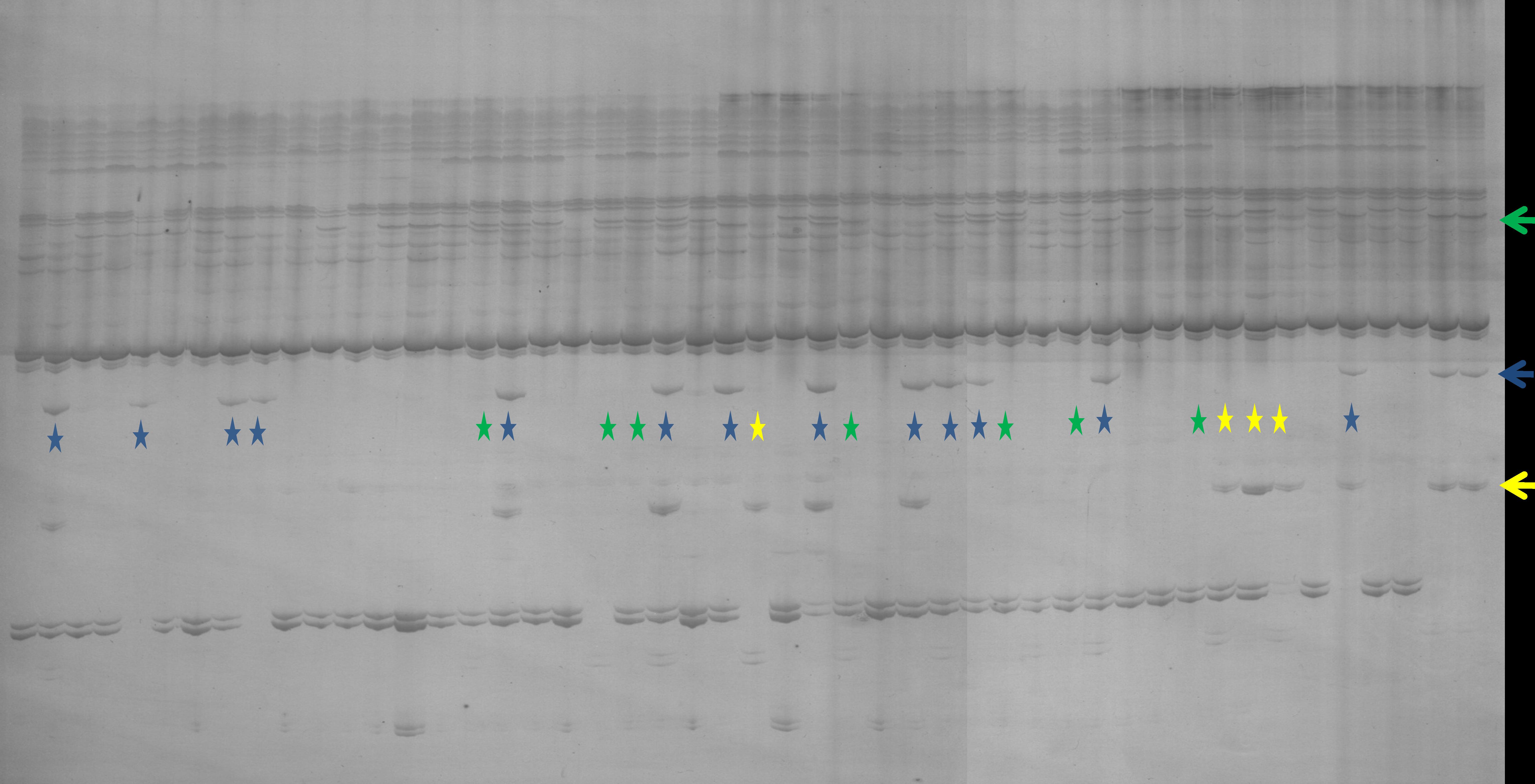

Supplement: FIGURE S1 — Selection of hybrid E. curvula plants with RAPD markers. Fragments were amplified with primer 248 and revealed in 6% acrylamide gels. Arrows indicate polymorphisms between the parental plants and the stars show offsprings harboring paternal markers. [file Image_1.JPEG]

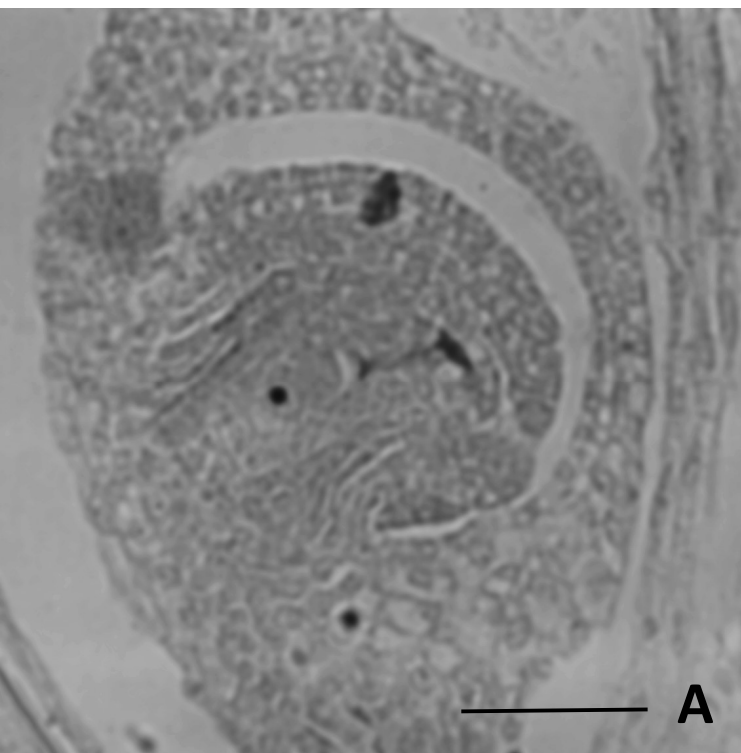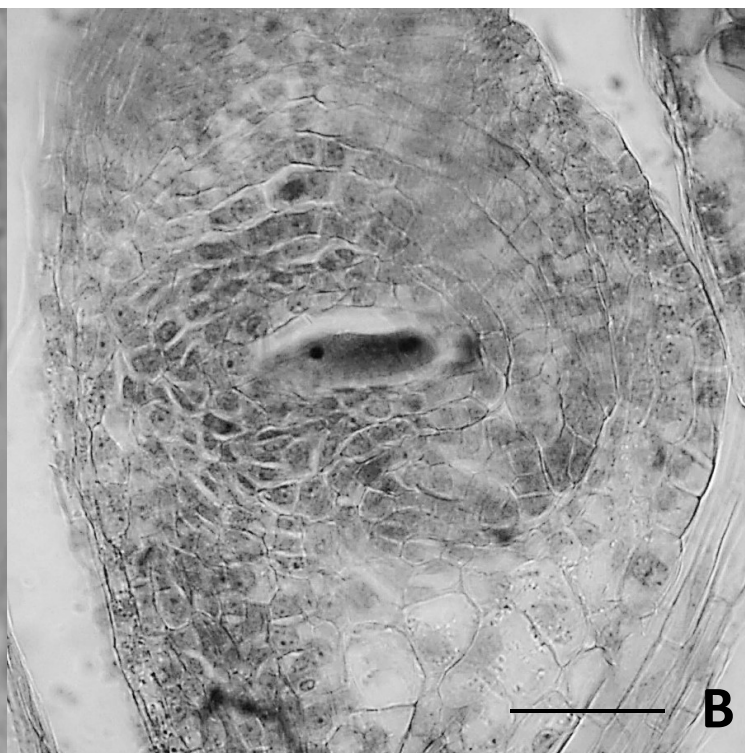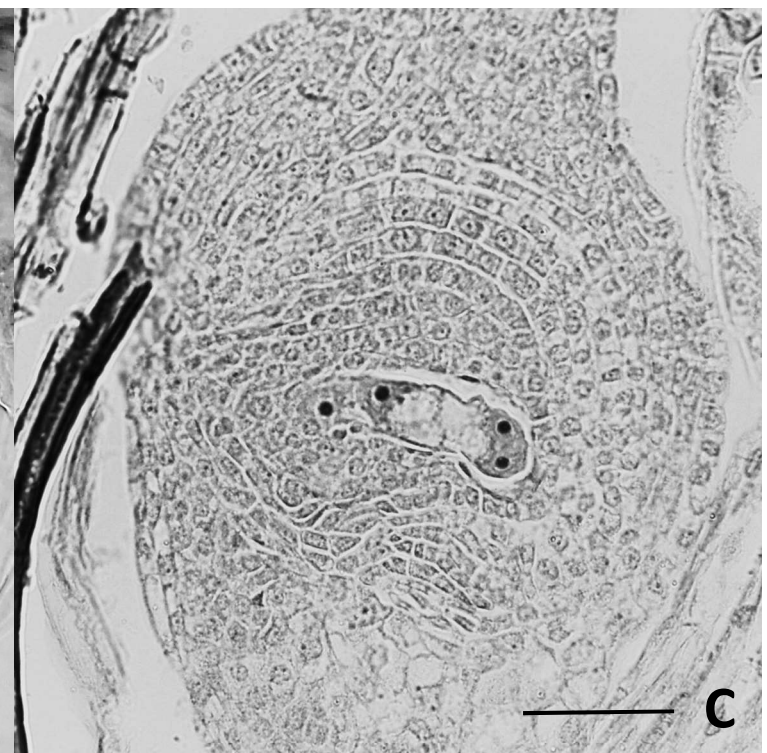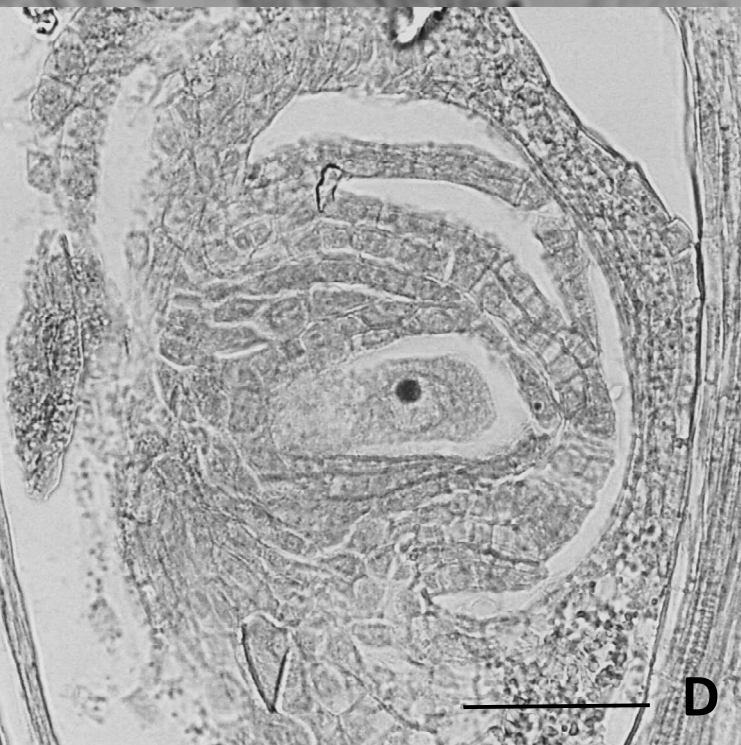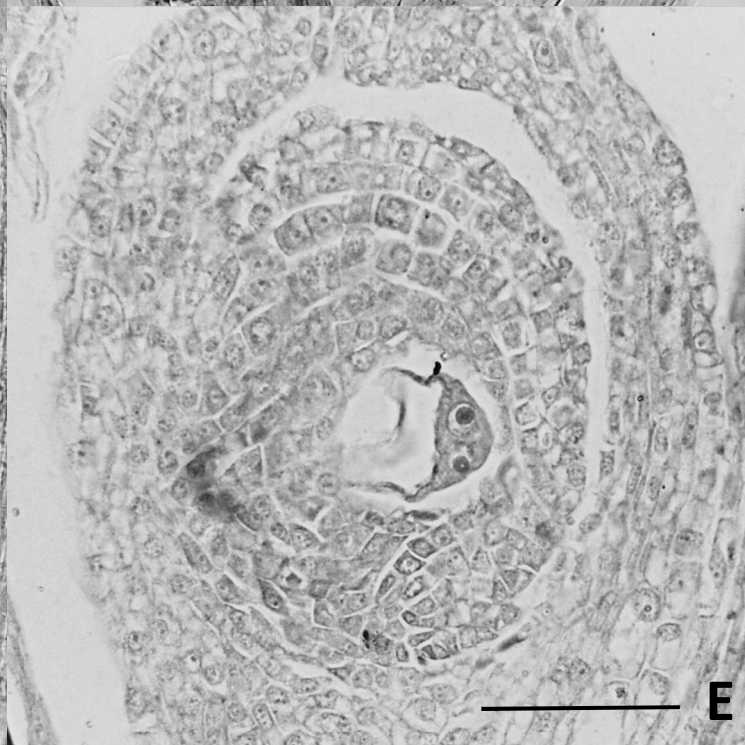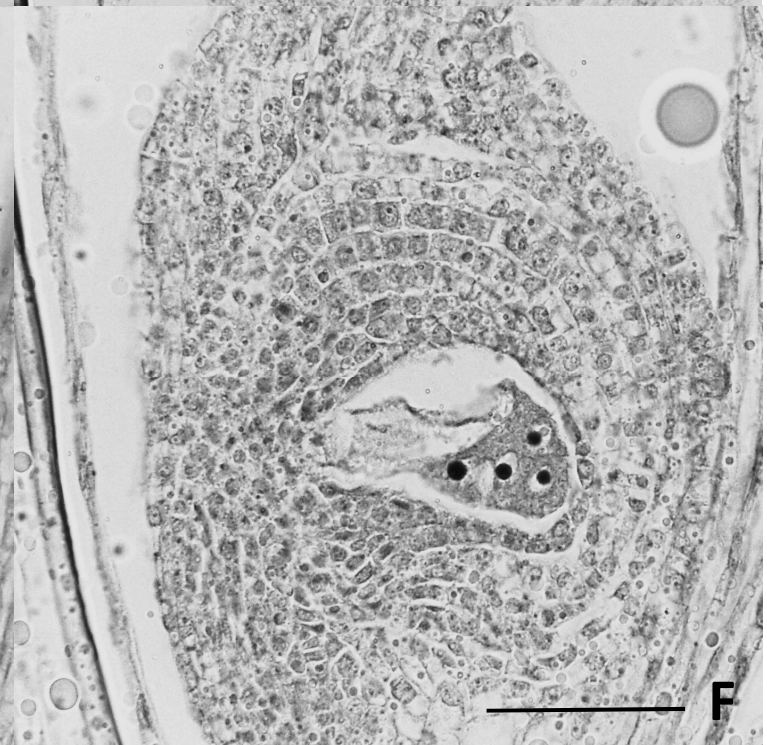

Supplement: FIGURE S2 — Development of the sexual (A–C) and diplosporic embryo sacs (D–F) in plants of weeping lovegrass. Bar: 50 μm. Sections dyed with safranina-fast green. (A) Megaspore mother cell and degenerated megaspores, (B) Binucleated embryo sac, (C) Tetranucleate embryo sac, (D) Elongated megaspore mother cell, (E) Binucleate embryo sac, (F) Tetranucleate embryo sac. [file Image_2.pdf]

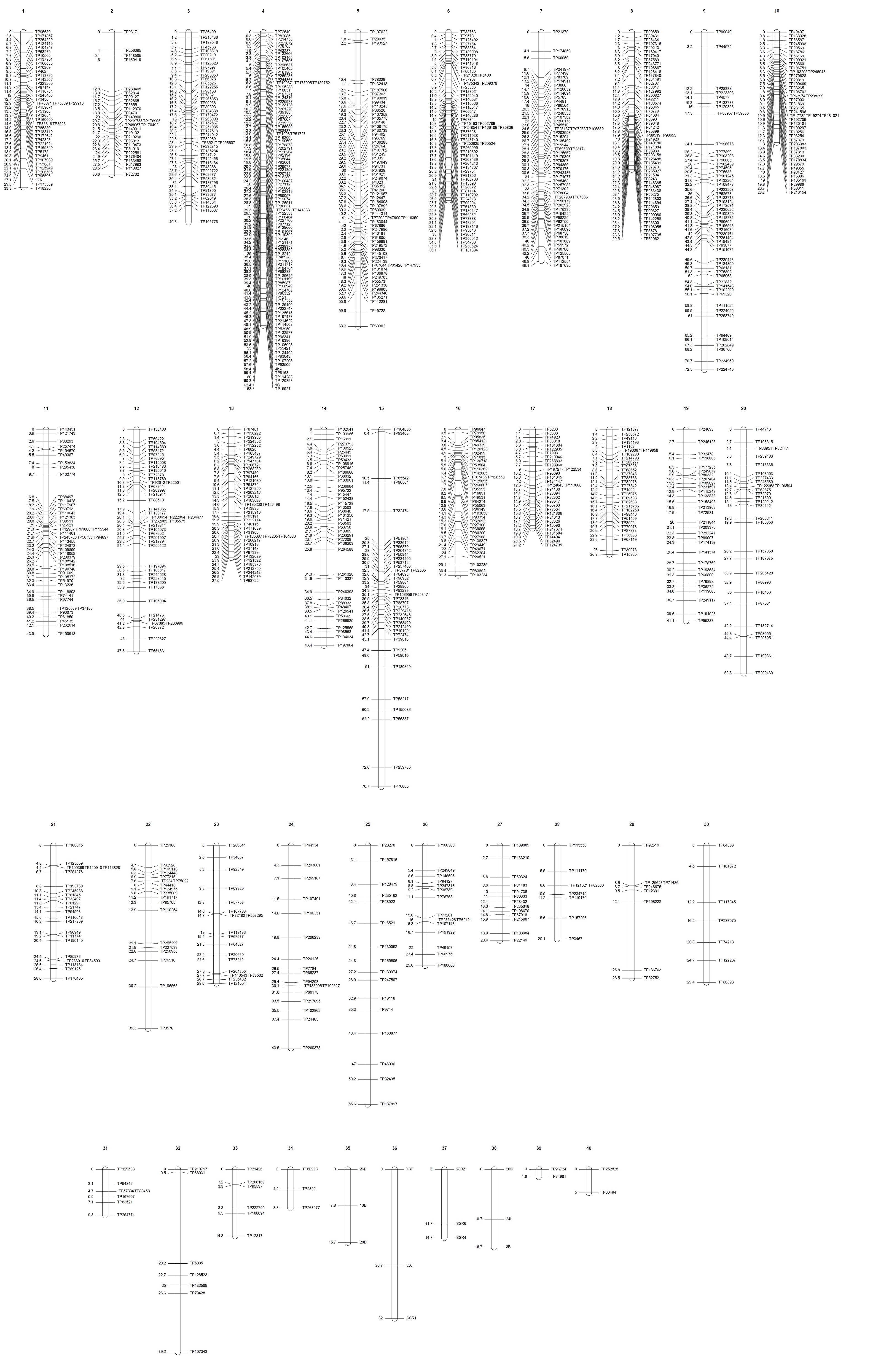

Supplement: FIGURE S3 — Linkage groups of the sexual plant OTA-S (E. curvula) obtained using GBS-SNPs, SSRs, and AFLPs. Marker positions are expressed in centimorgans. [file Image_3.jpg]

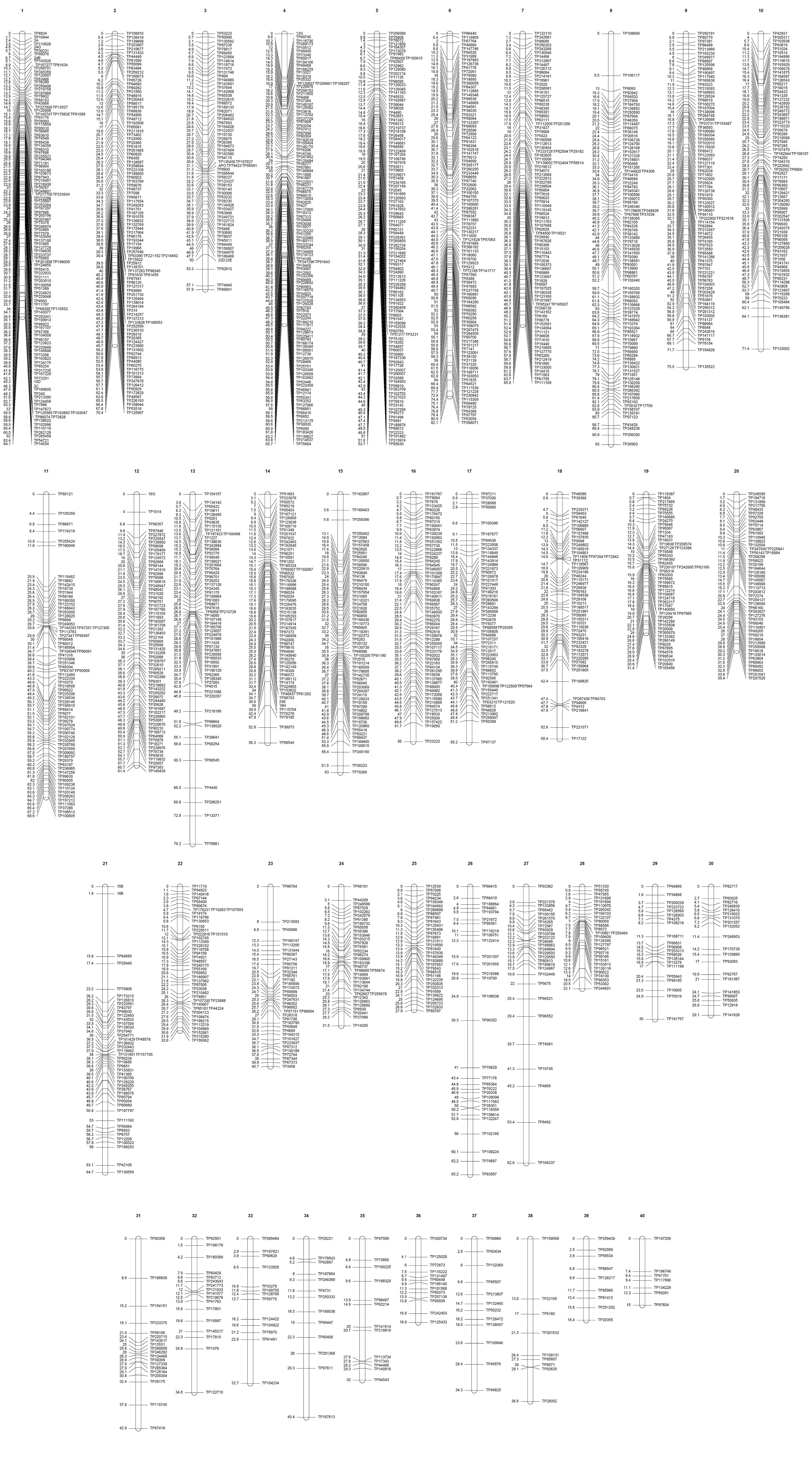

Supplement: FIGURE S4 — Linkage groups of the facultative apomictic plant Don Walter (E. curvula) obtained using GBS-SNPs, SSRs, and AFLPs. Marker positions are expressed in centimorgans. [file Image_4.jpg]
