# Supplementary material for: A High-Density Linkage Map of the Forage Grass Eragrostis curvula and Localization of the Diplospory Locus
Source: Front Plant Sci. 2019 Jul 12;10:918. doi: 10.3389/fpls.2019.00918 (PMC6640543; doi:10.3389/fpls.2019.00918)
Supplement: FIGURE S5 — Linkage groups from the facultative apomictic cv. Don Walter (E. curvula) showing the QTLs positions (in cM) for diplospory. The LOD threshold to consider a QTL as significant is indicated as a dashed line at a LOD value of 3.9. [file Image_5.pdf]

Group 1

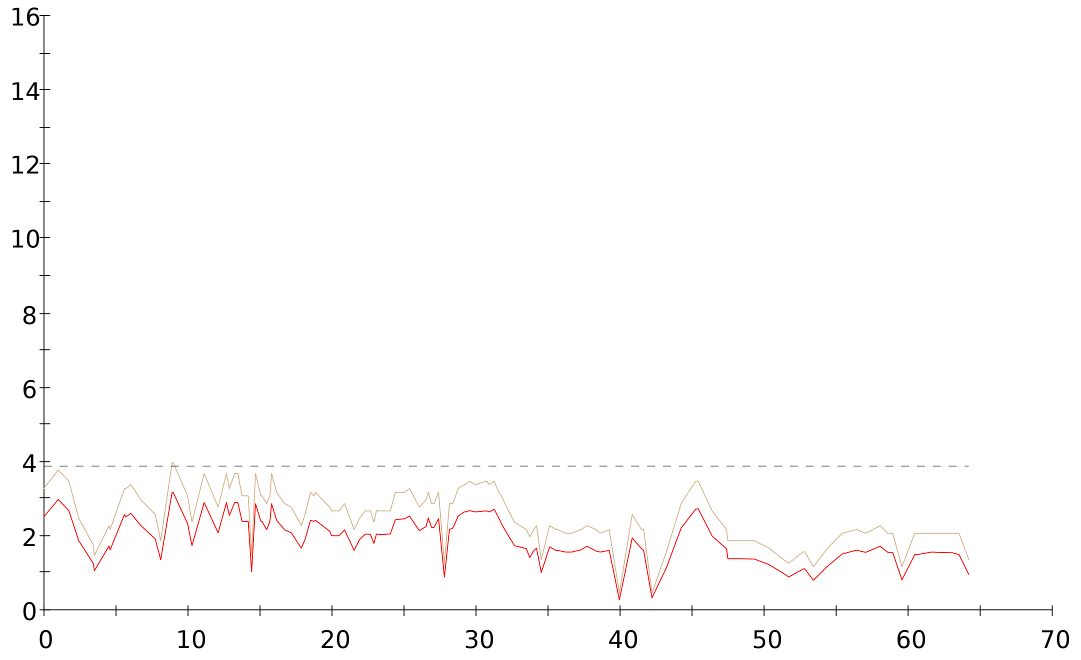

Group 10

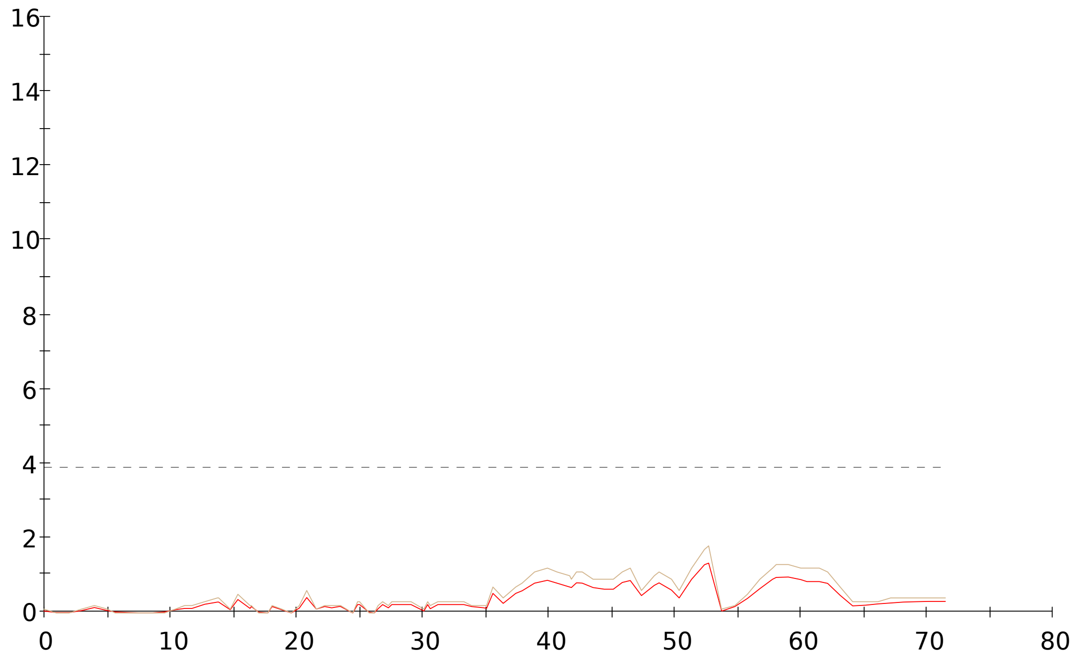

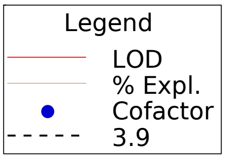

Group 11

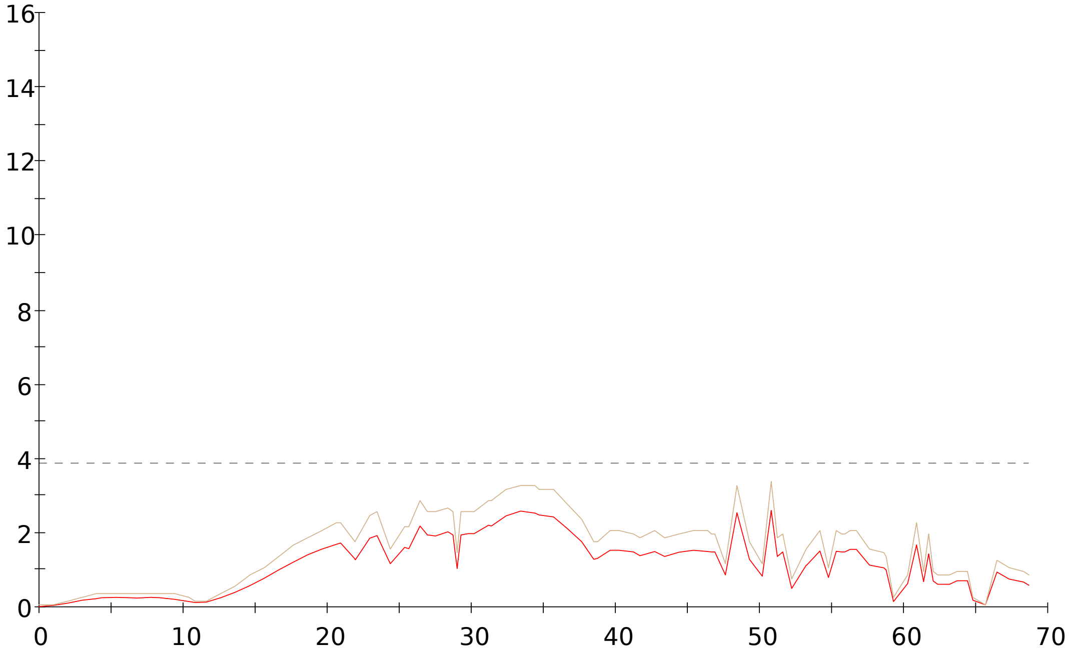

Group 12

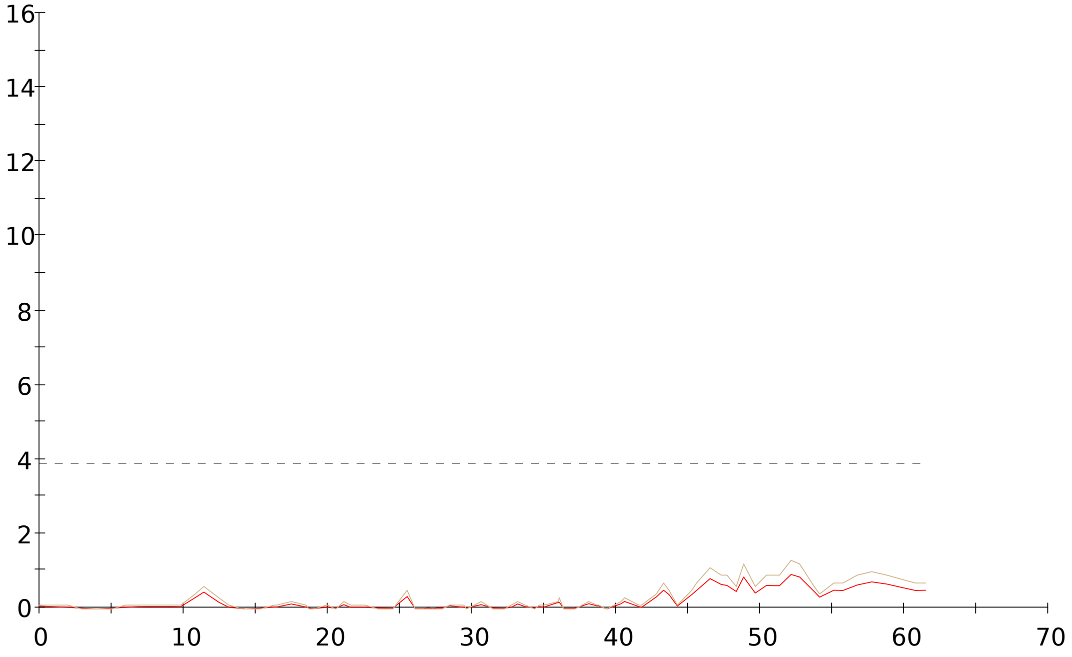

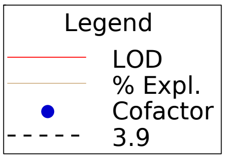

Group 13

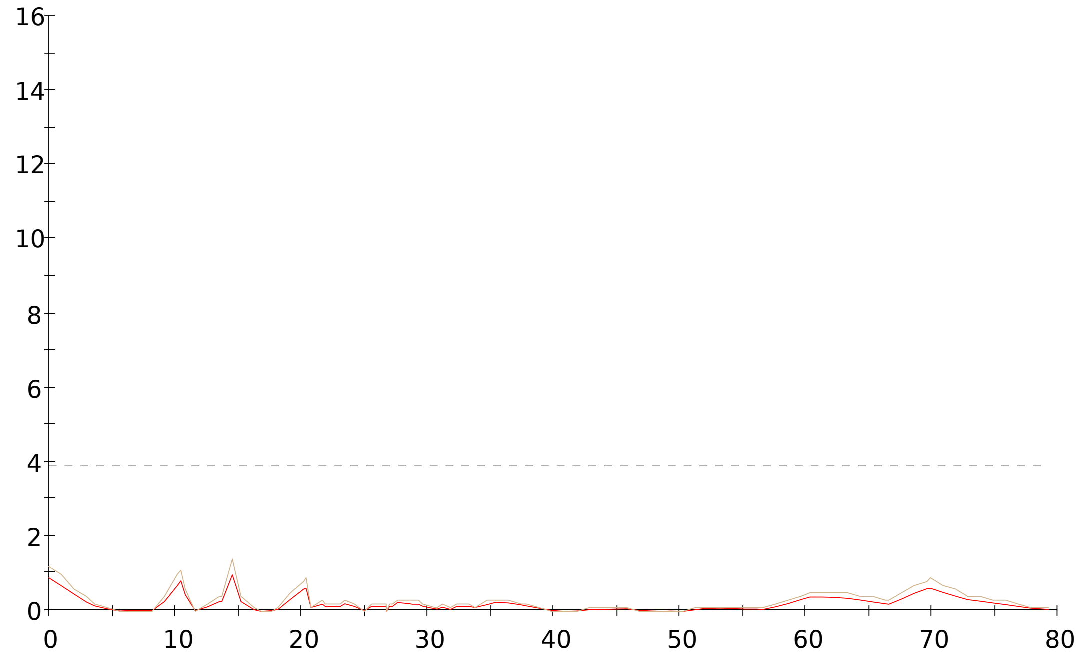

Group 14

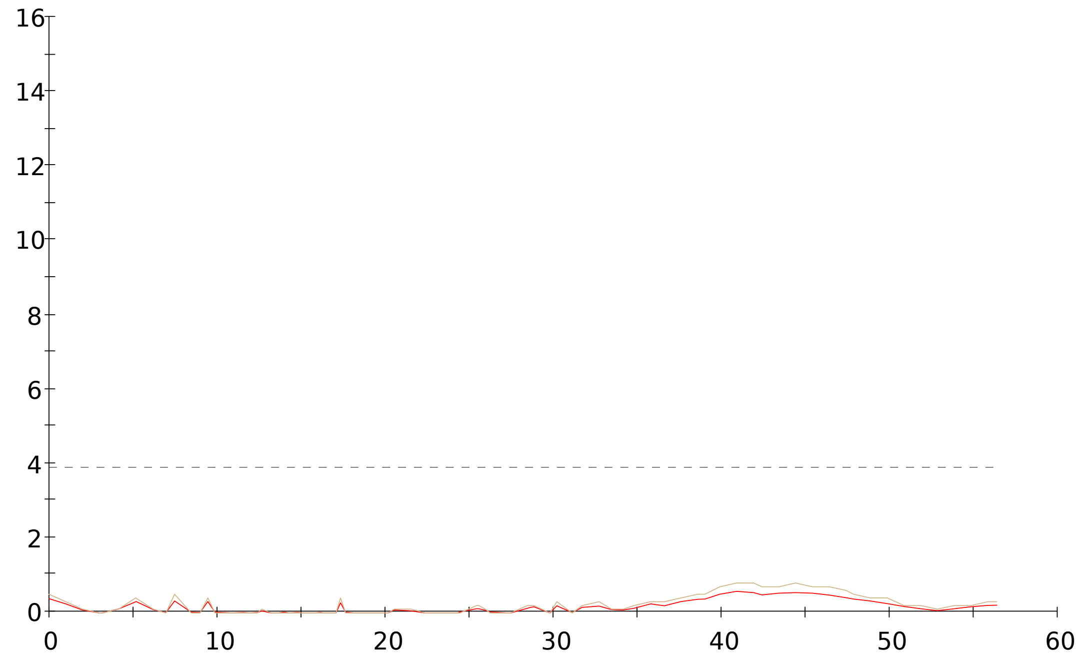

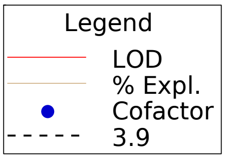

Group 13

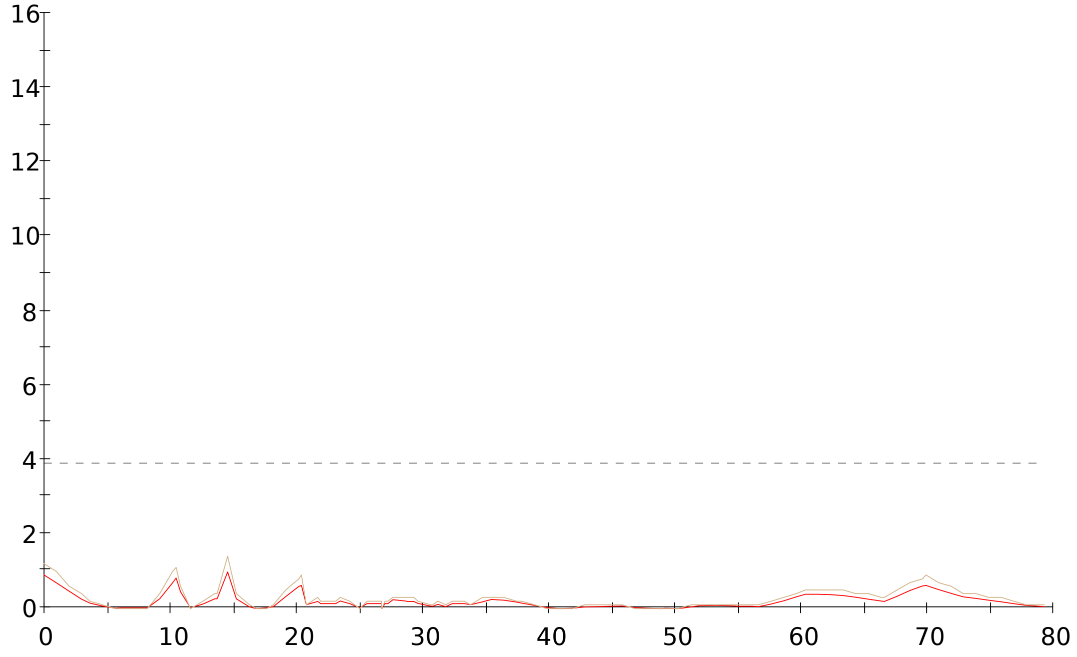

Group 14

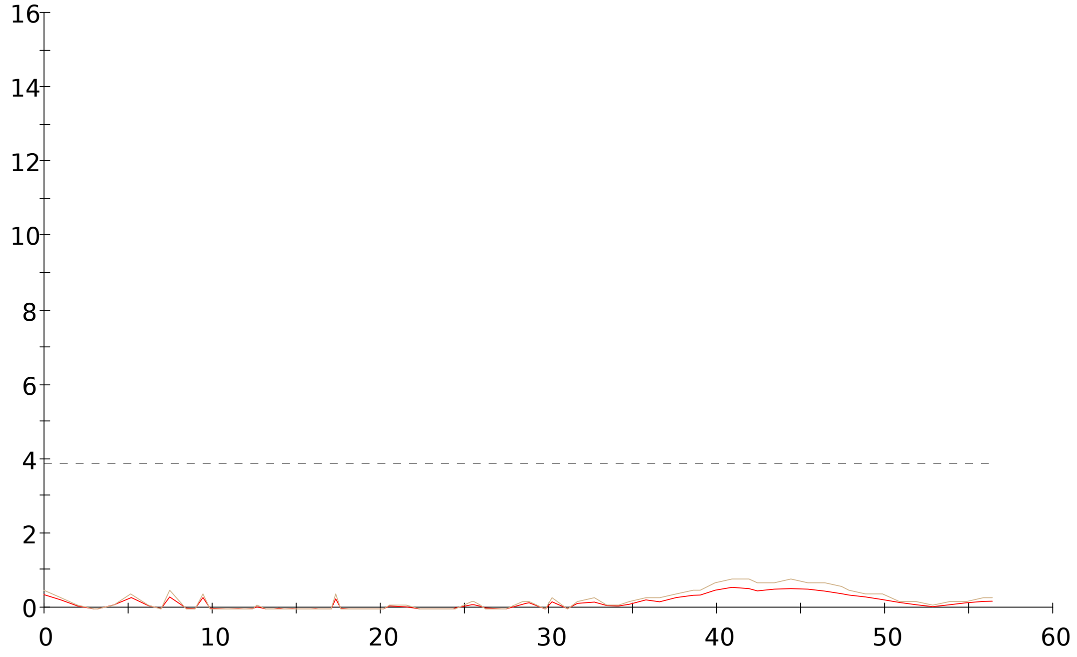

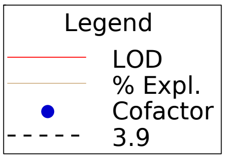

Group 15

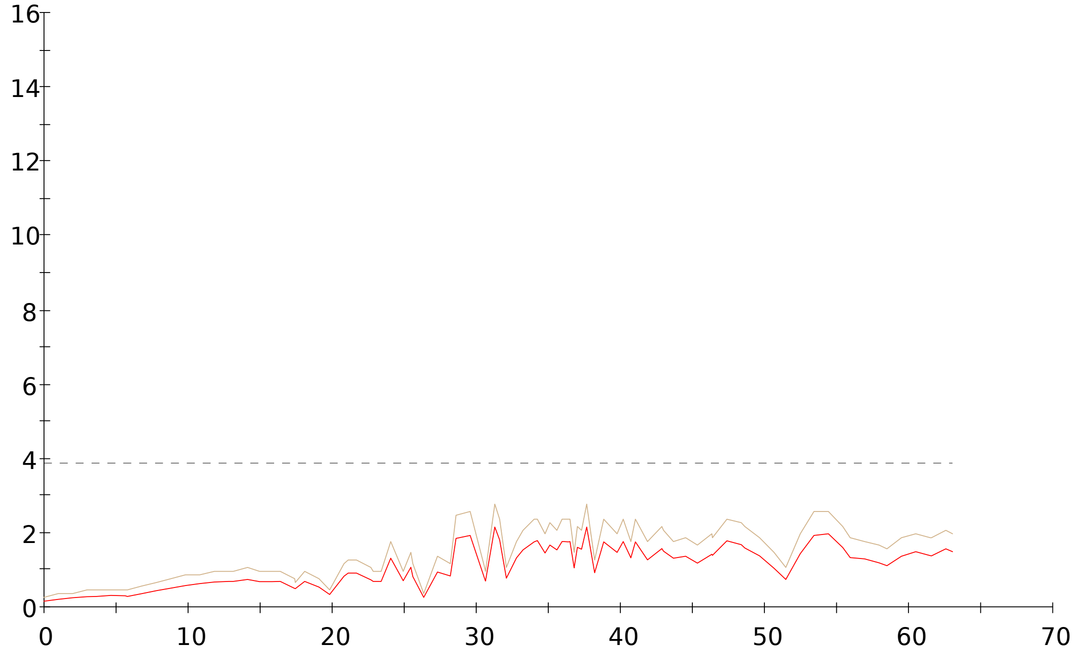

Group 16

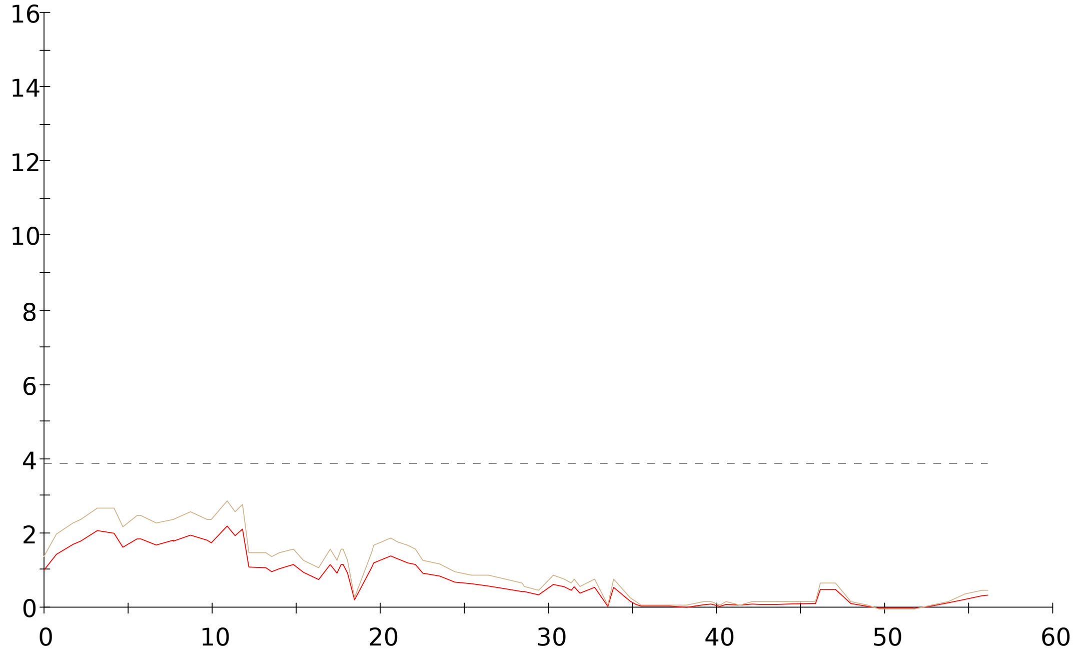

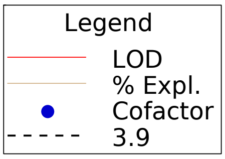

Group 17

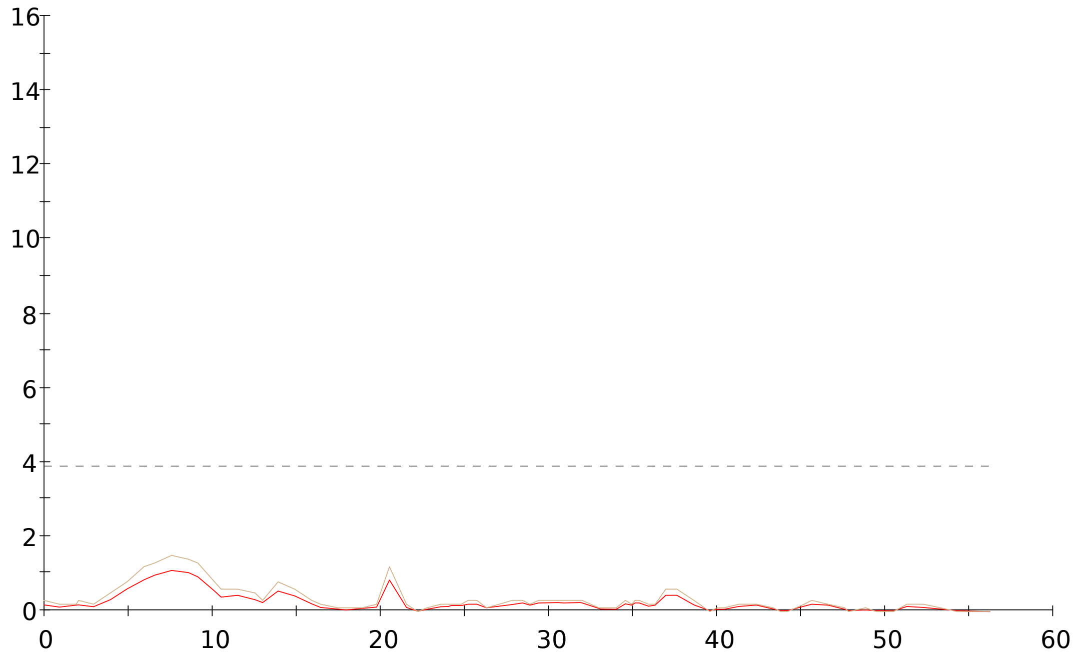

Group 18

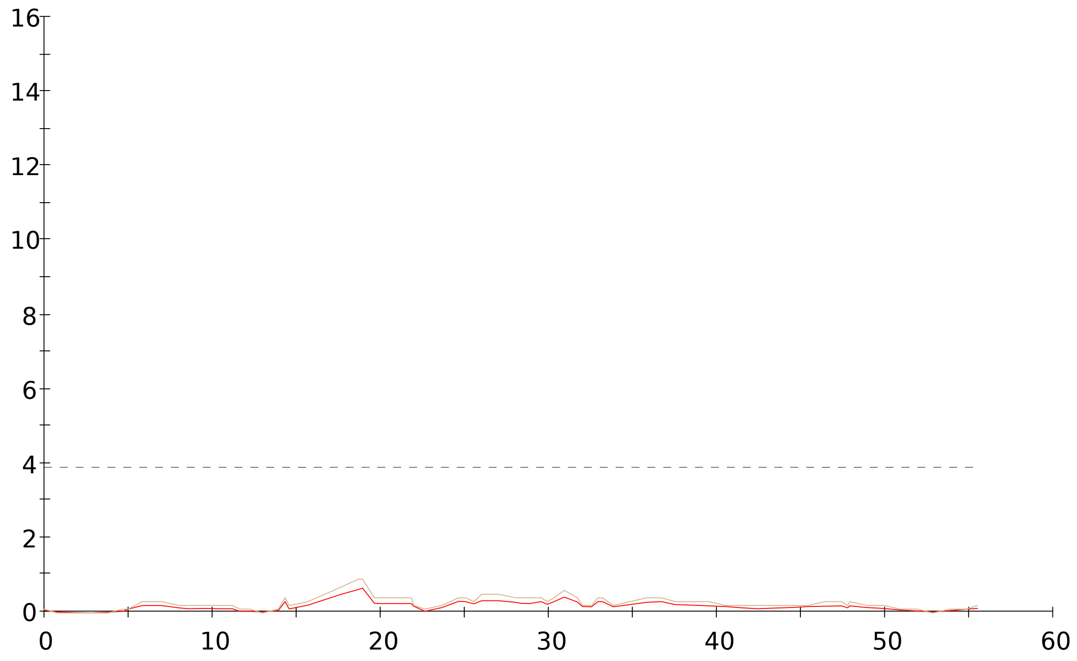

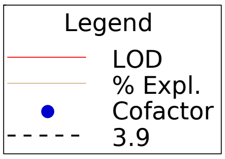

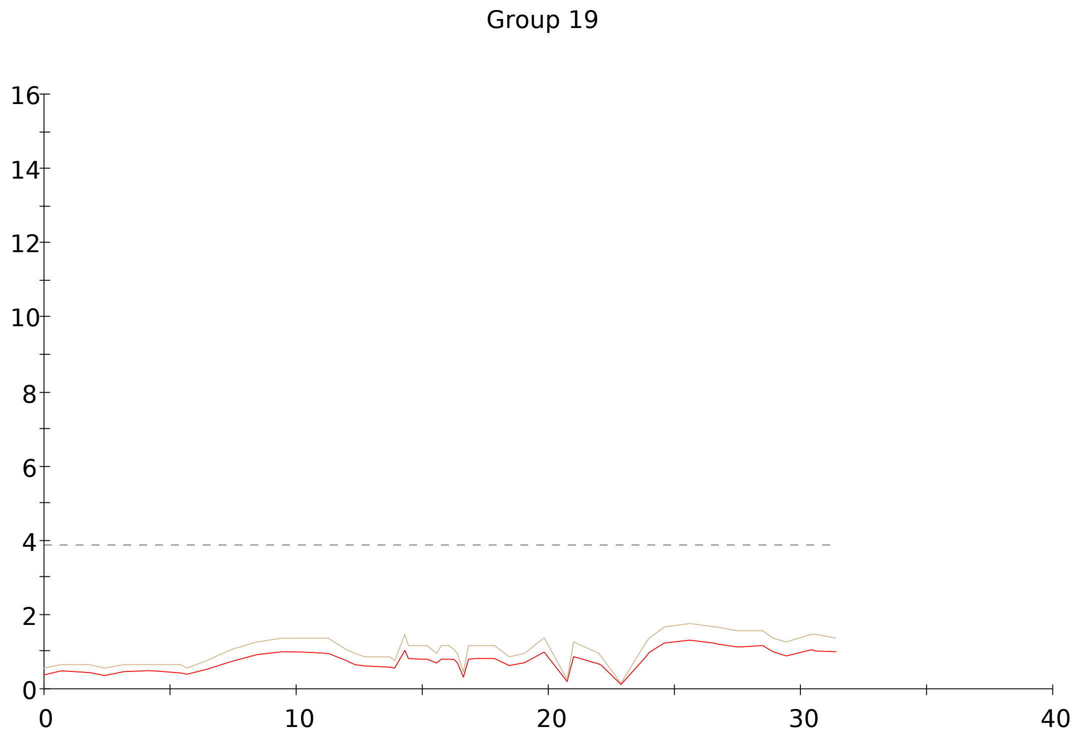
Group 19
16
14
12
10
8
6
4
2
0
0
10
20
30
40

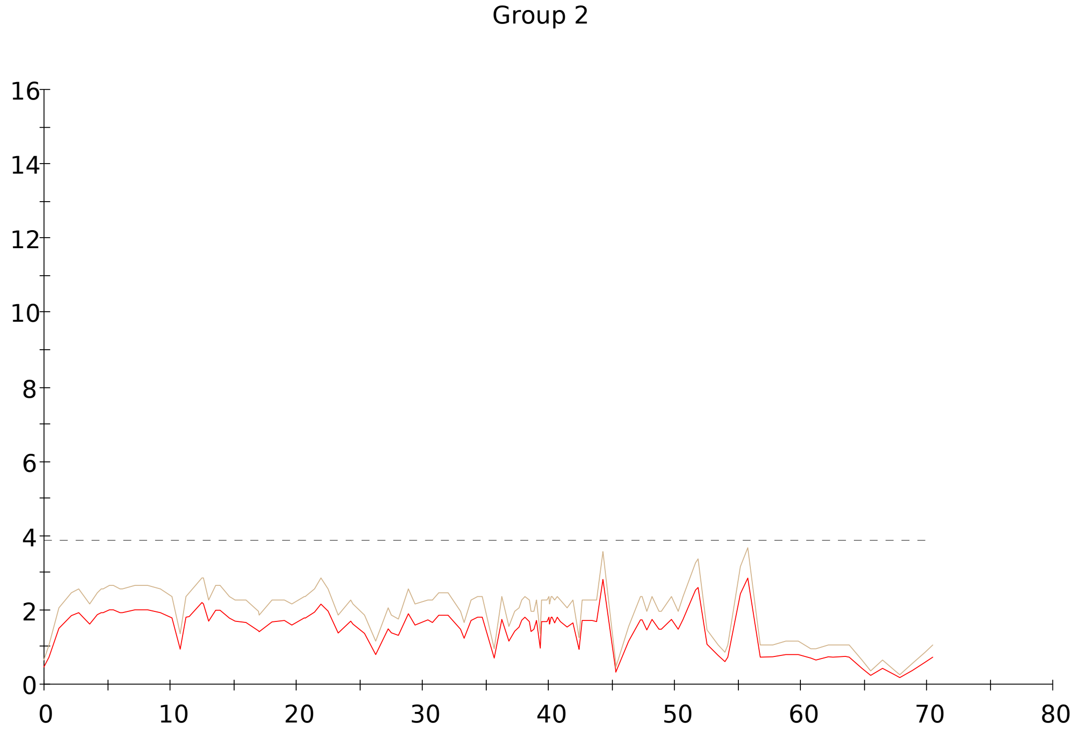
Group 2
16
14
12
10
8
6
4
2
0
0
10
20
30
40
50
60
70
80

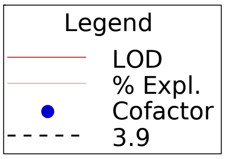
Legend
LOD
% Expl.
Cofactor
3.9

Group 20

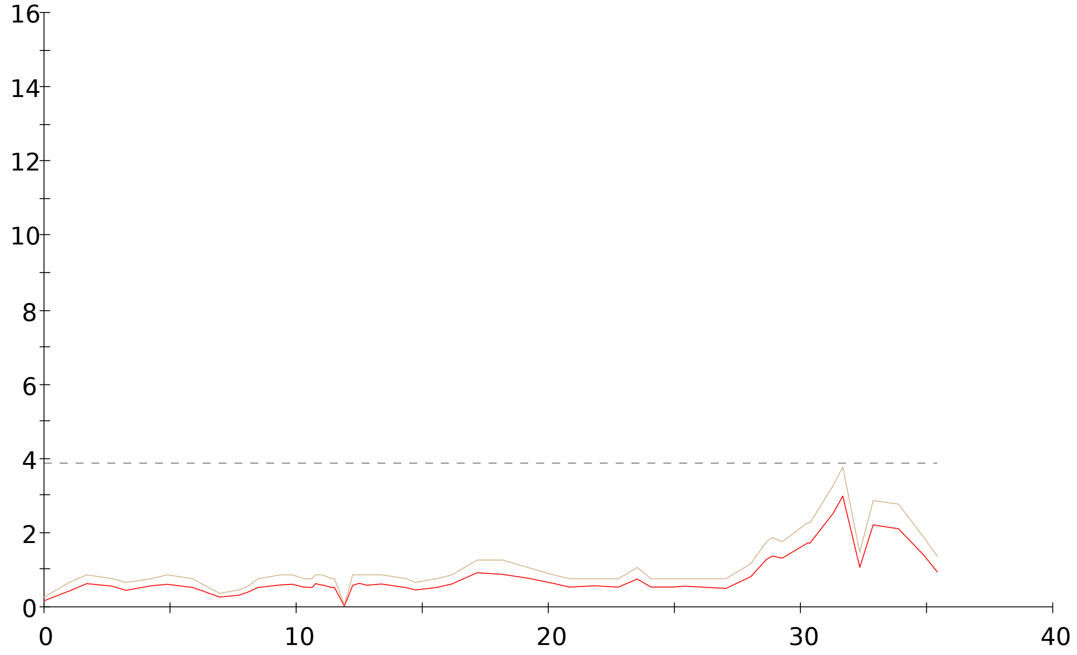

Group 21

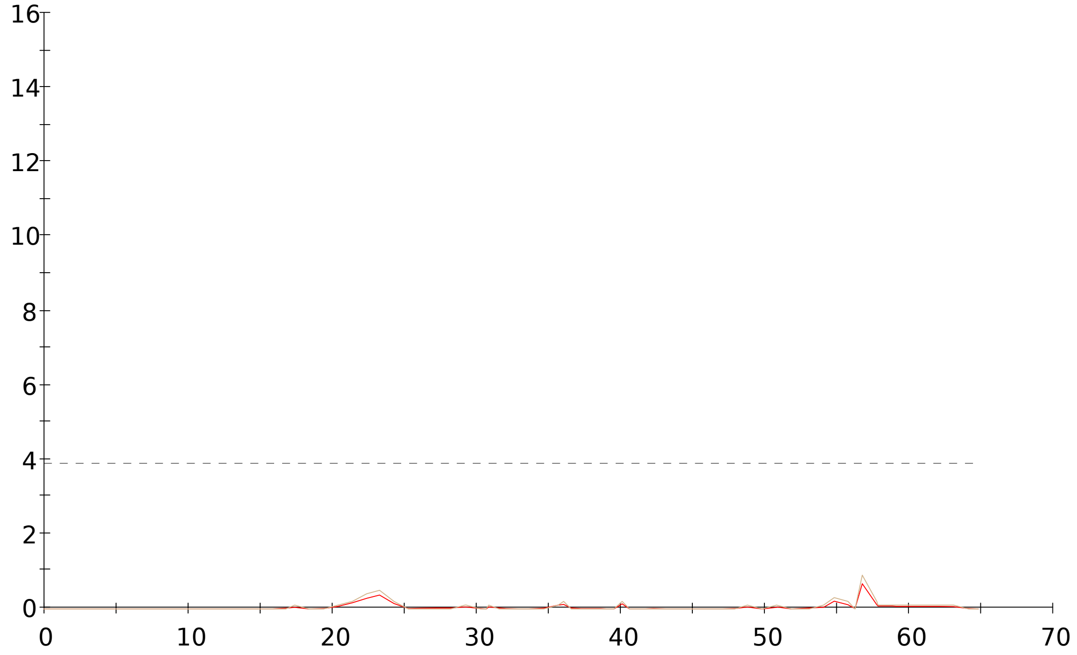

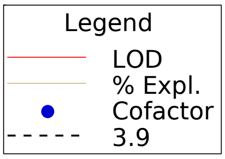

Group 22

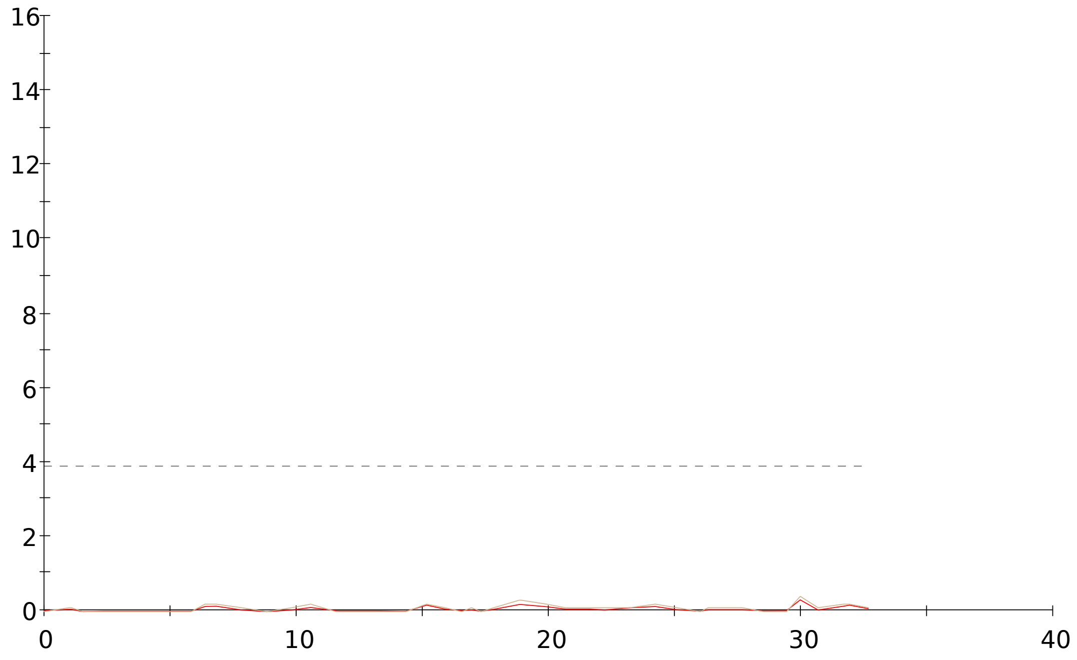

Group 23

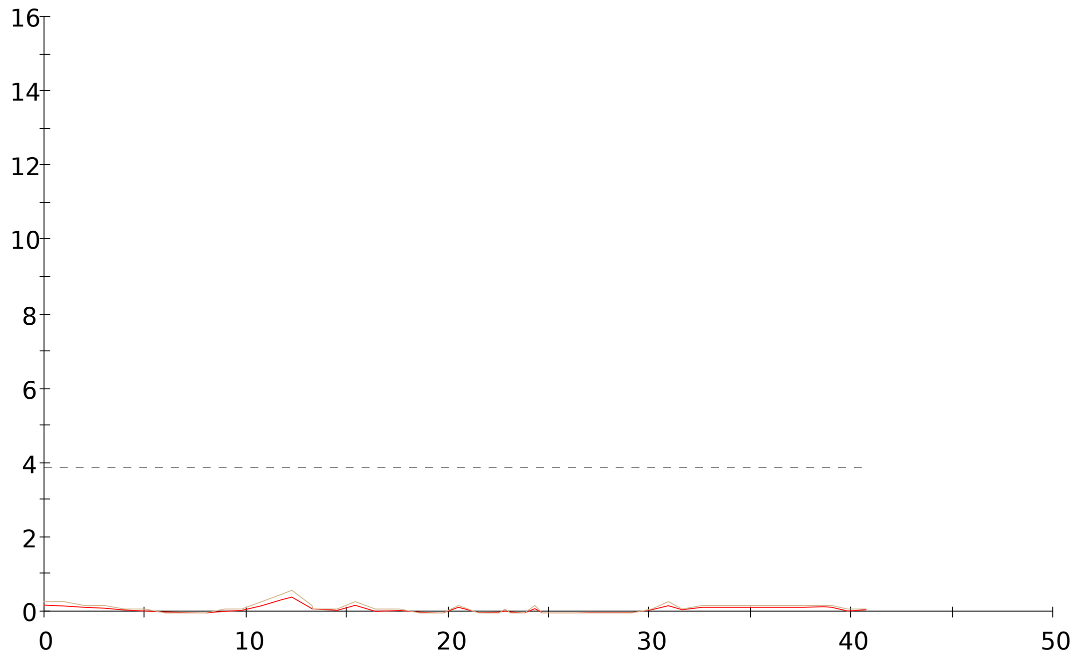

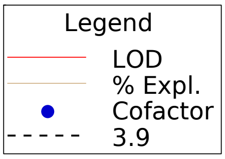

Group 24

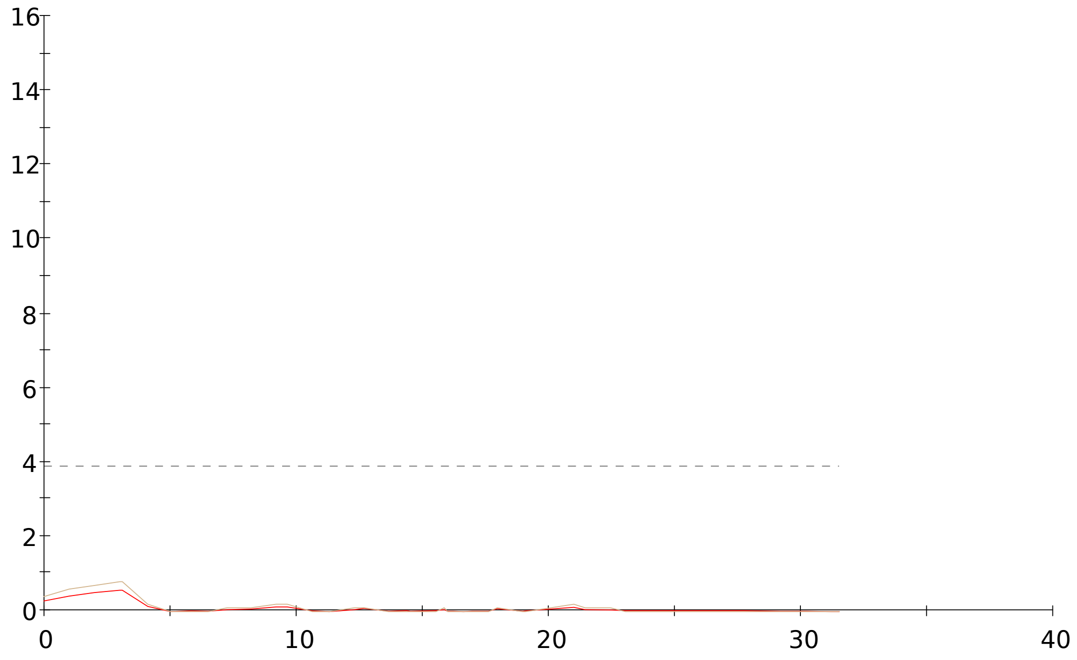

Group 25

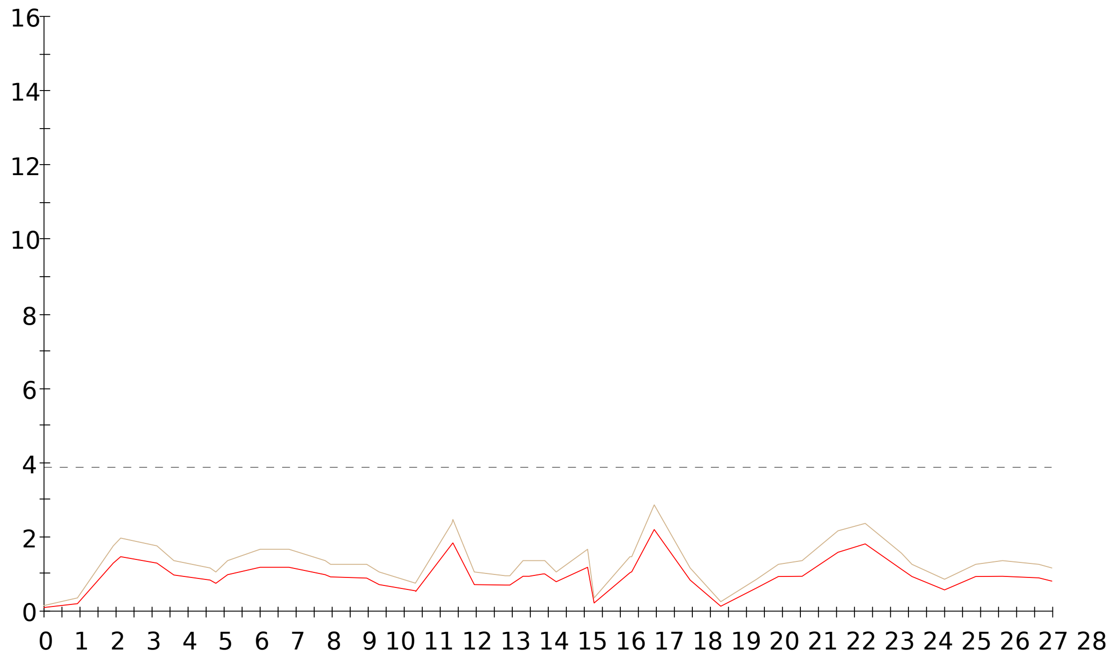

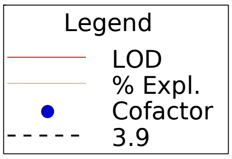

Group 26

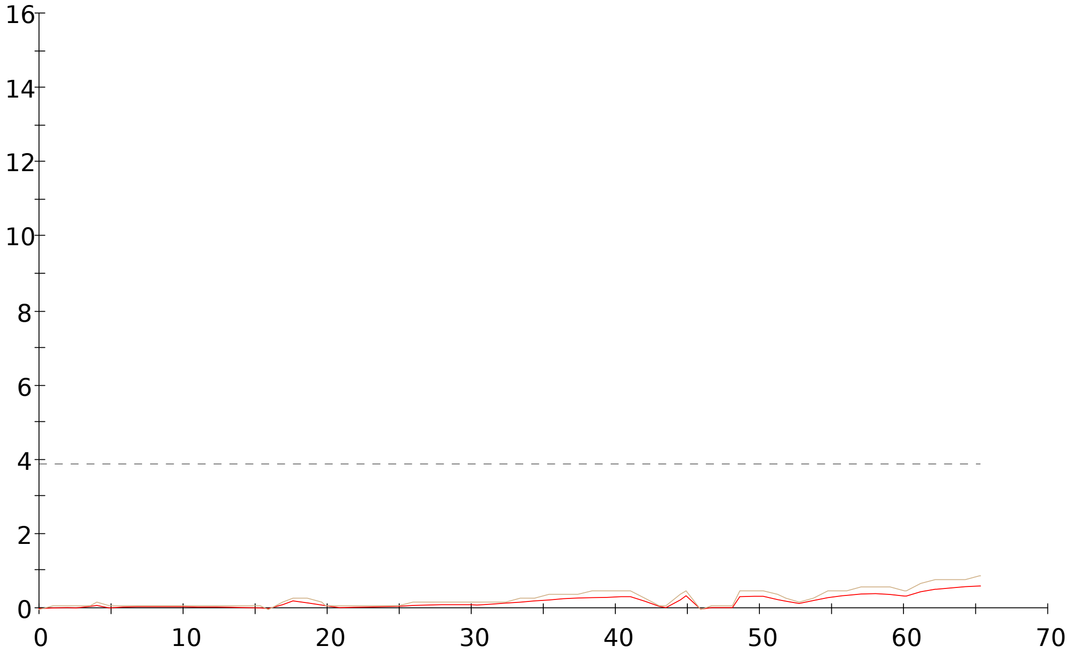

Group 27

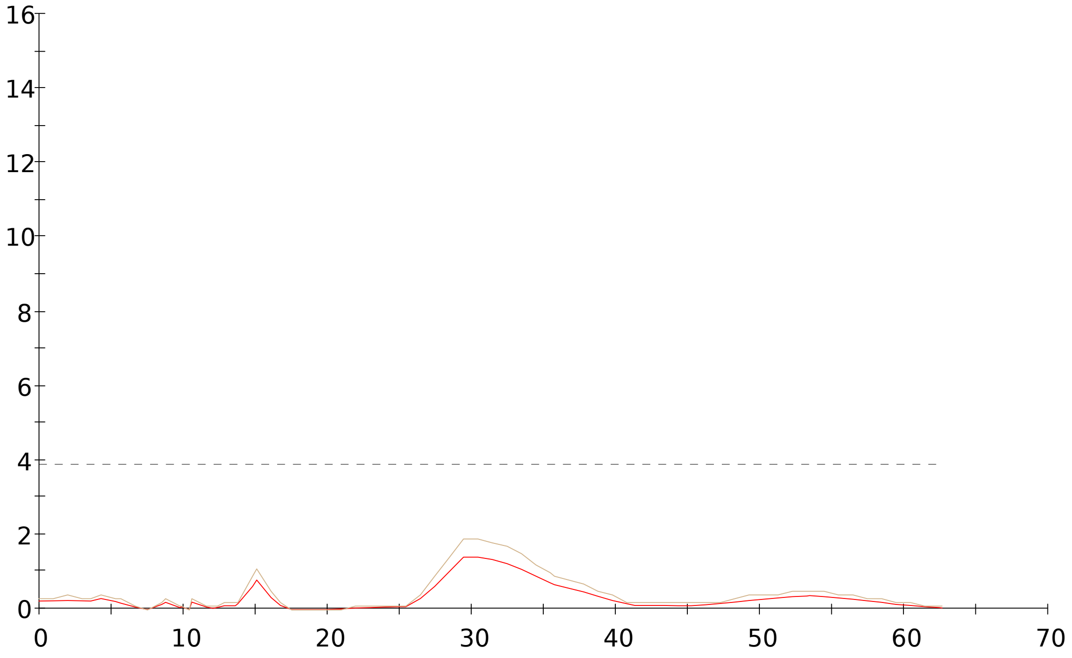

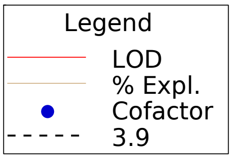

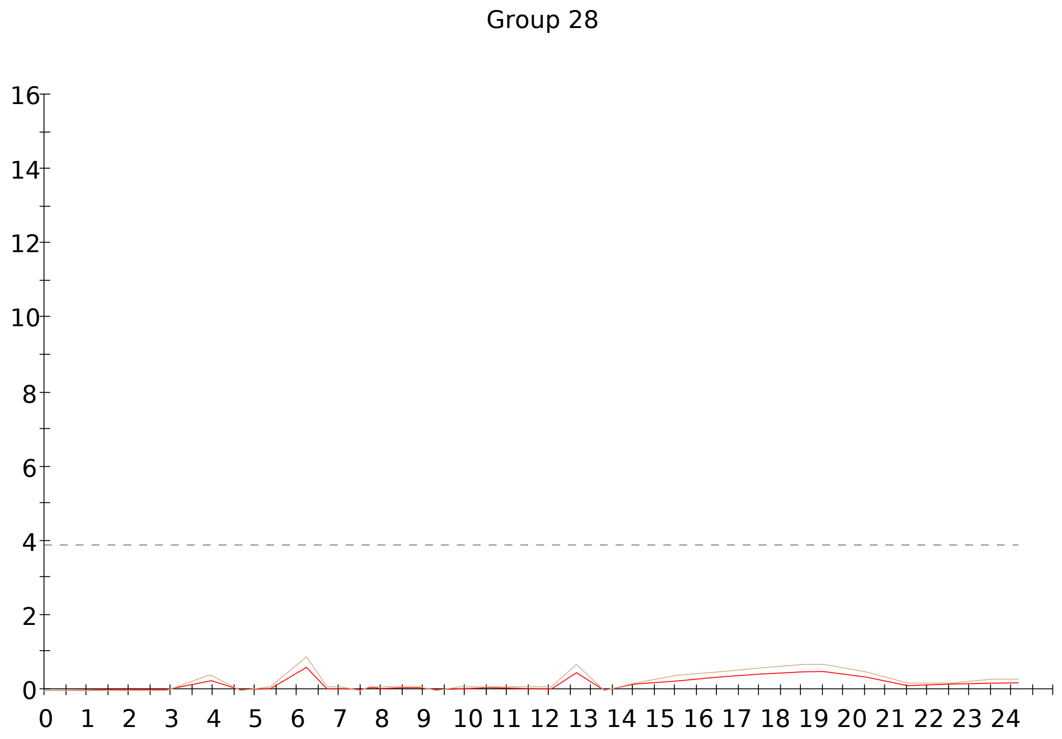
Group 28
16
14
12
10
8
6
4
2
0
0 1 2 3 4 5 6 7 8 9 10 11 12 13 14 15 16 17 18 19 20 21 22 23 24

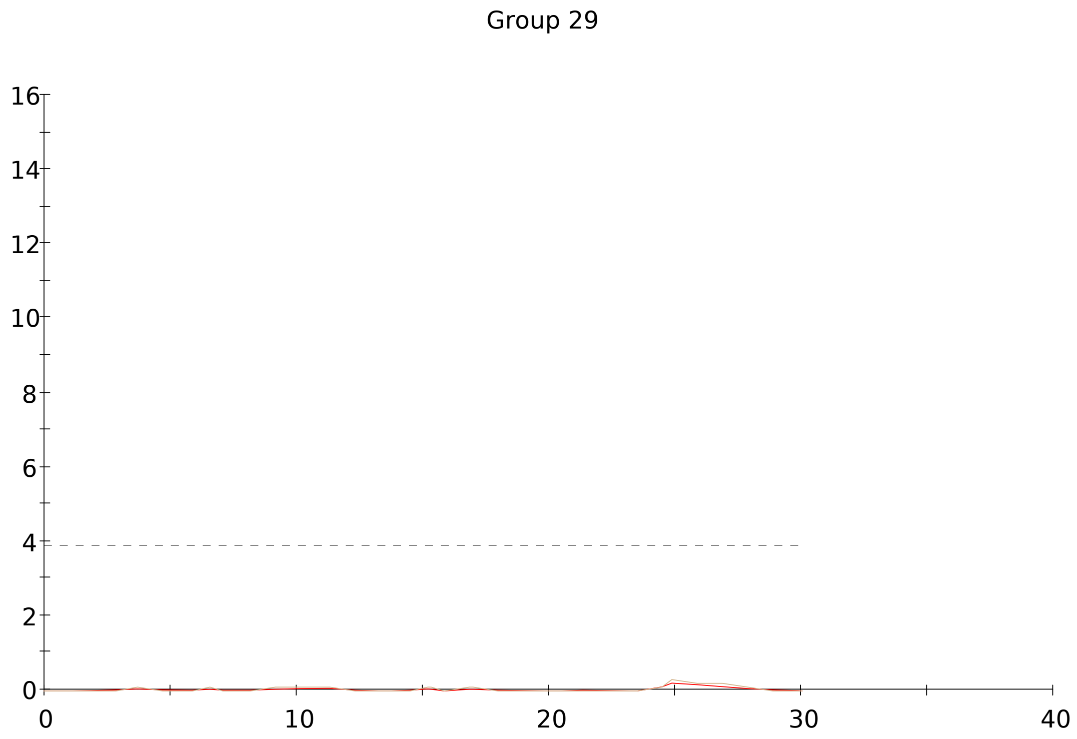
Group 29
16
14
12
10
8
6
4
2
0
0 10 20 30 40

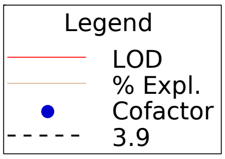
Legend
LOD
% Expl.
Cofactor
3.9

Group 3

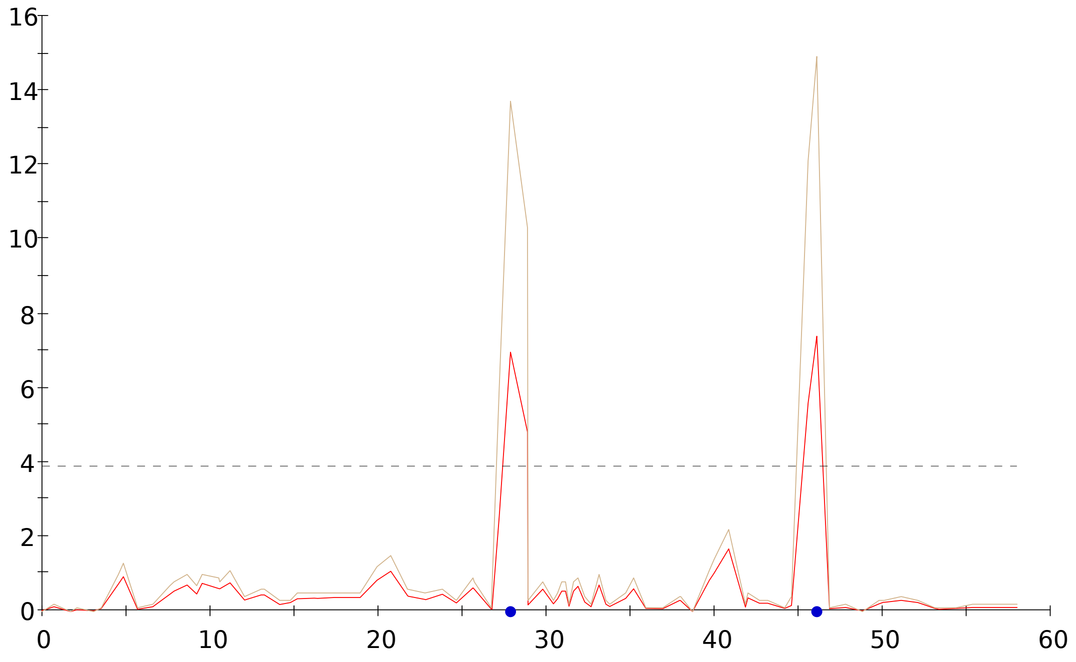

Group 30

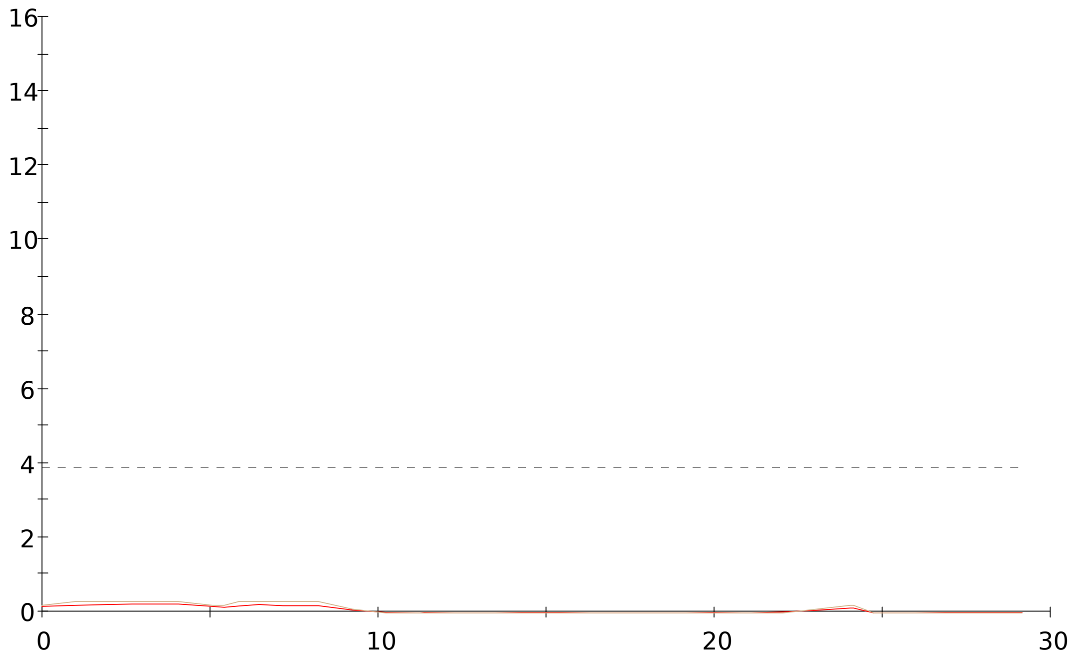

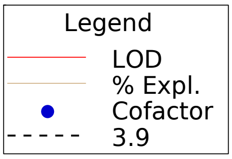

Group 31

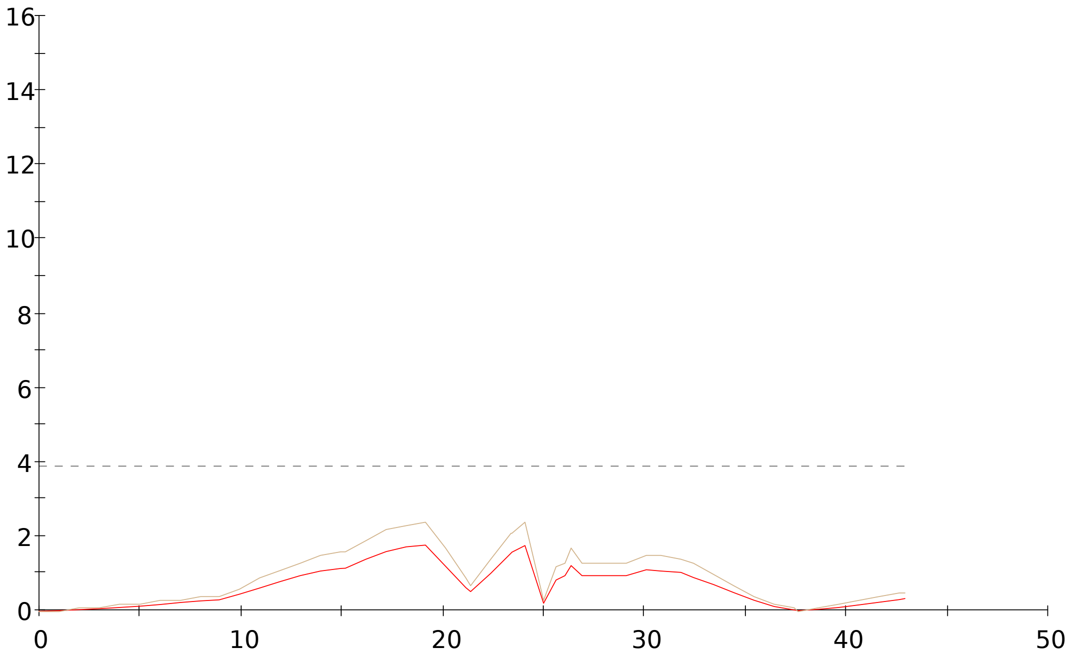

Group 32

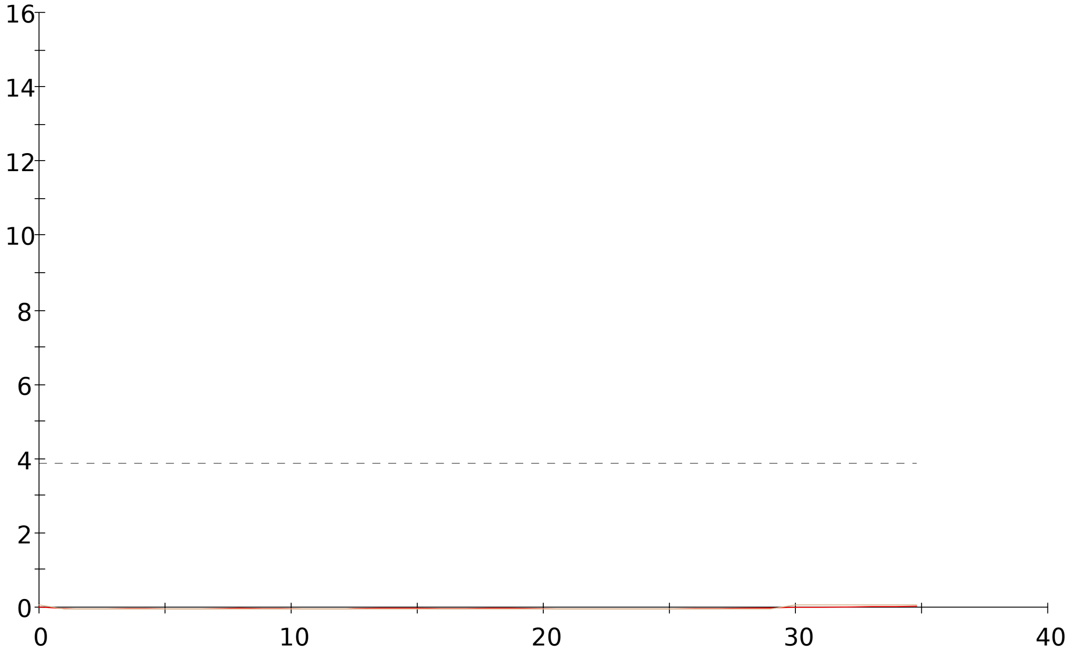

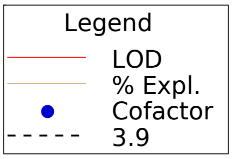

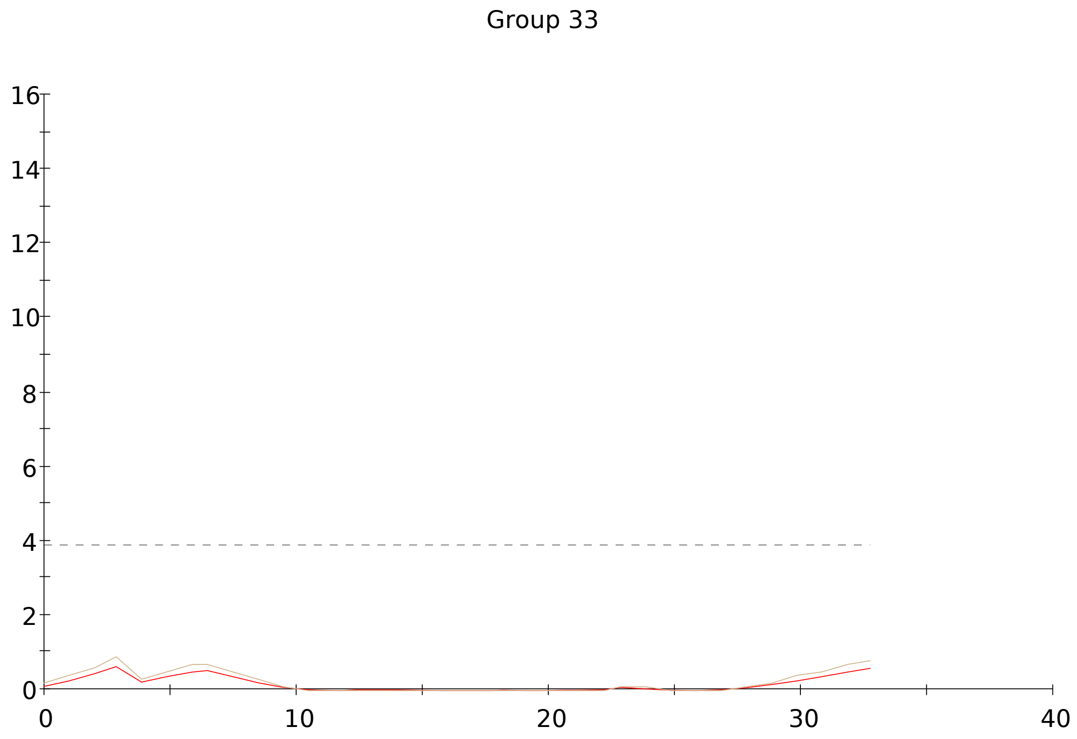
Group 33
16
14
12
10
8
6
4
2
0
0
10
20
30
40

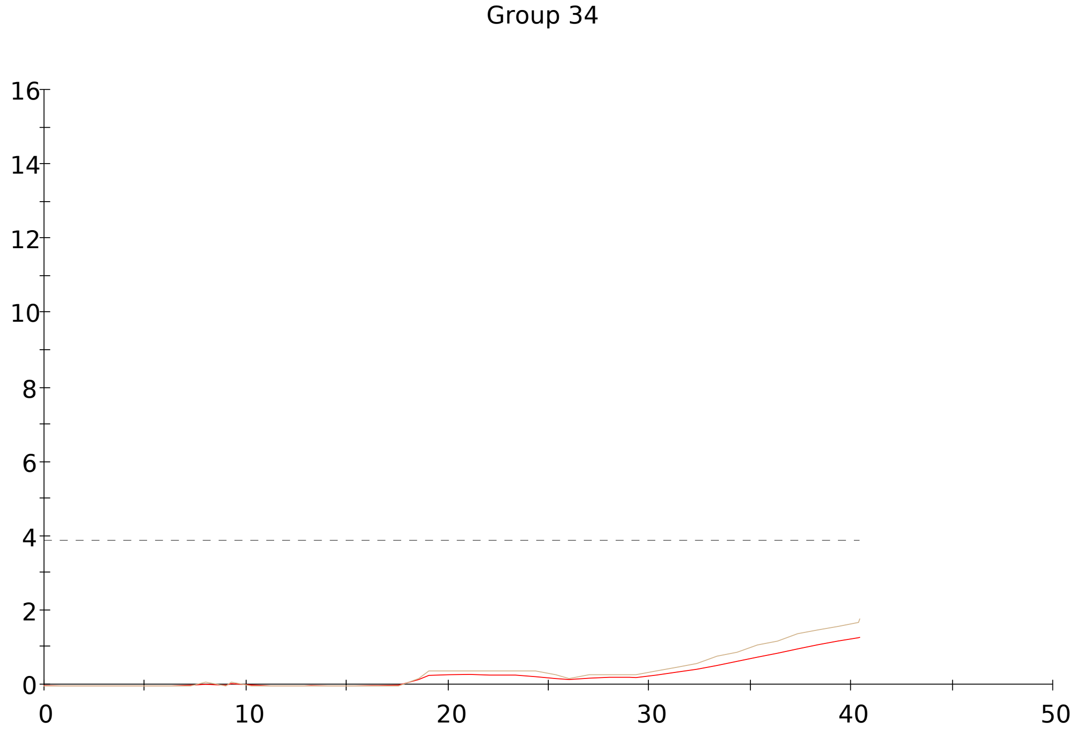
Group 34
16
14
12
10
8
6
4
2
0
0
10
20
30
40
50

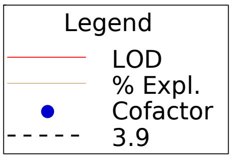
Legend
LOD
% Expl.
Cofactor
3.9

Group 35

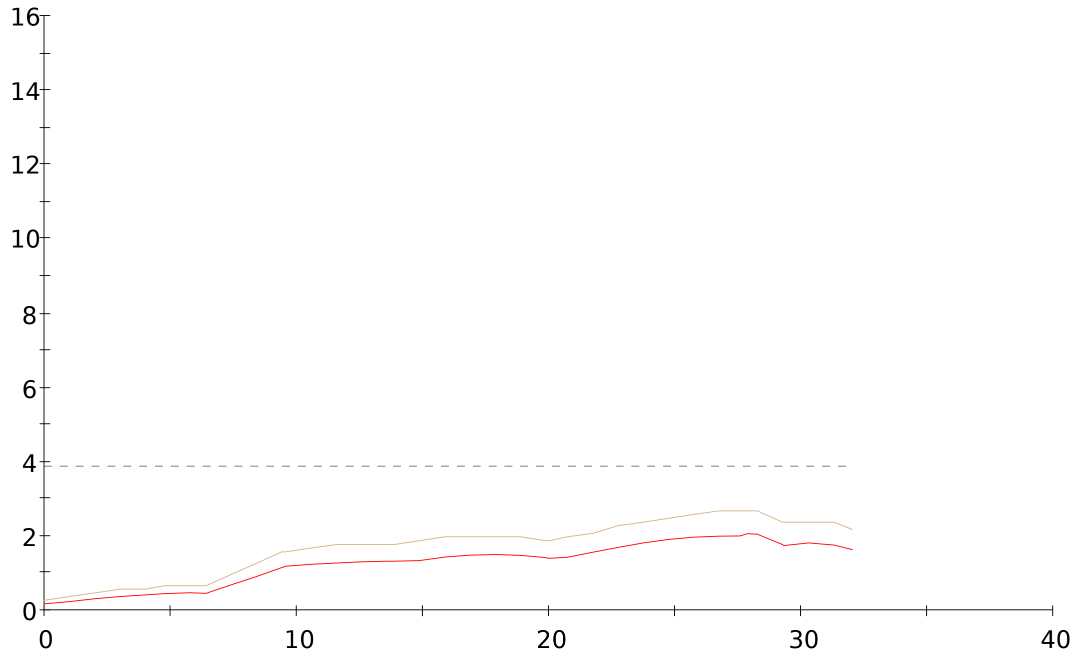

Group 36

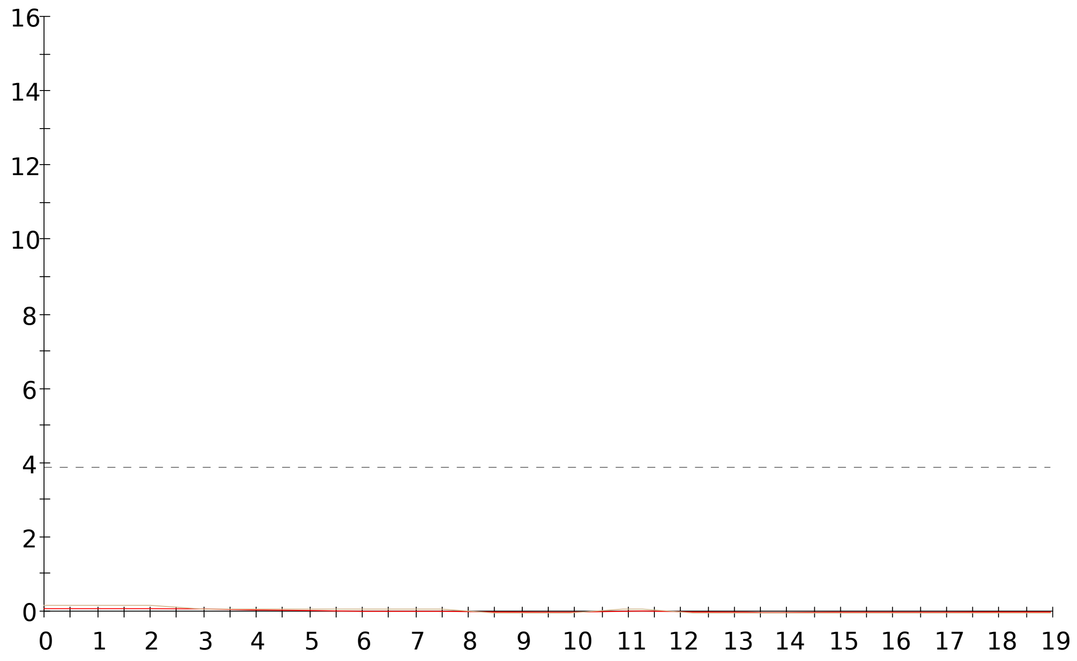

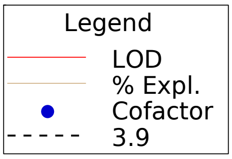

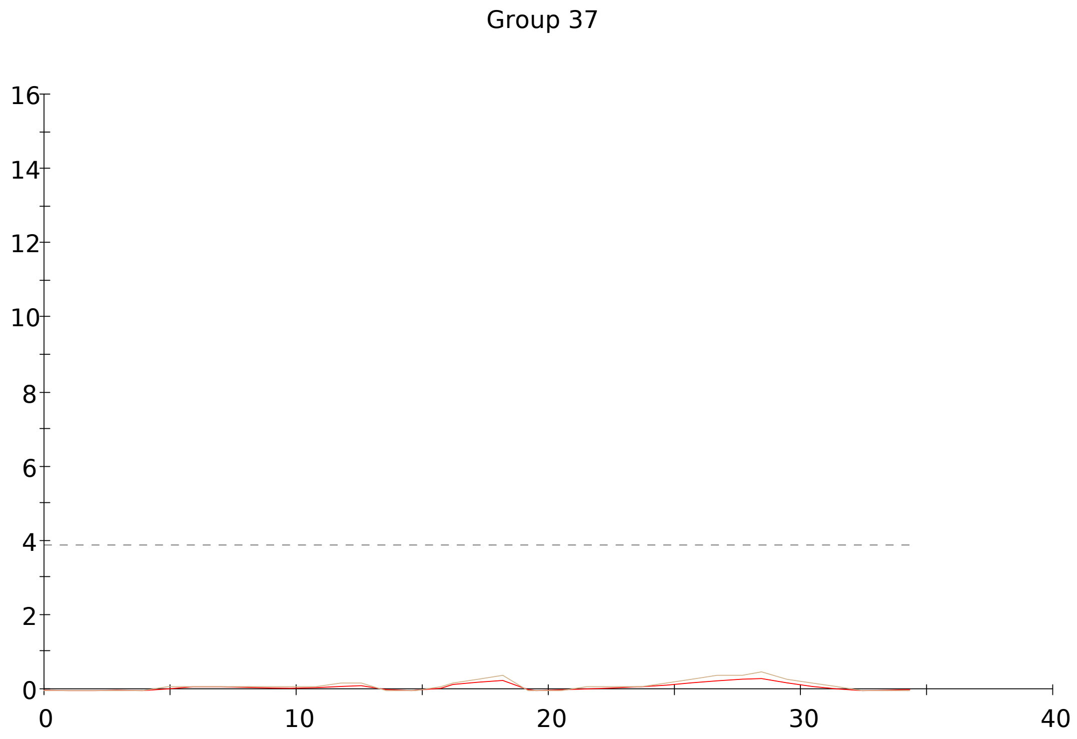
Group 37
16
14
12
10
8
6
4
2
0
0
10
20
30
40

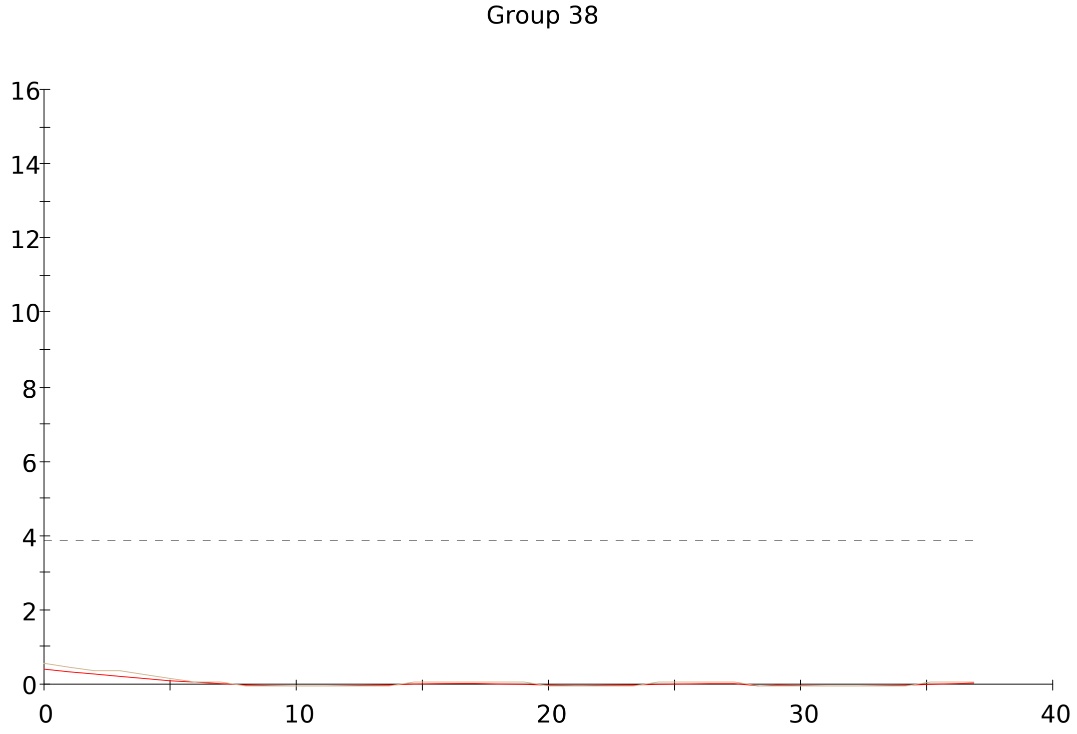
Group 38
16
14
12
10
8
6
4
2
0
0
10
20
30
40

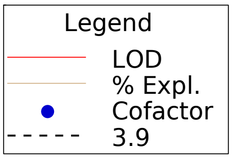
Legend
LOD
% Expl.
Cofactor
3.9

Group 39

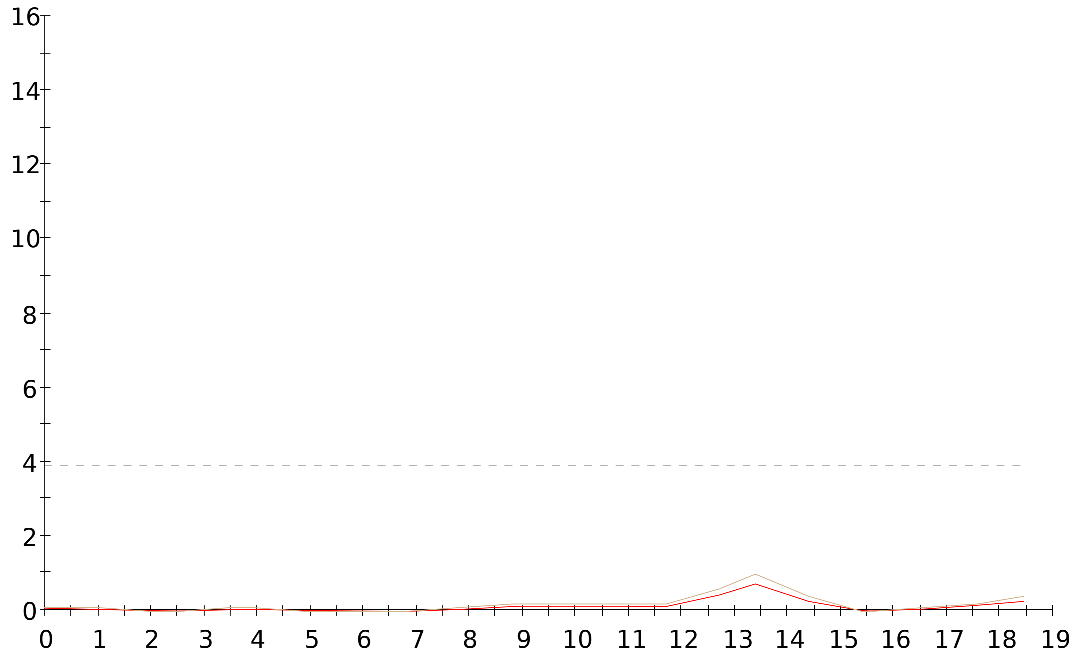

Group 4

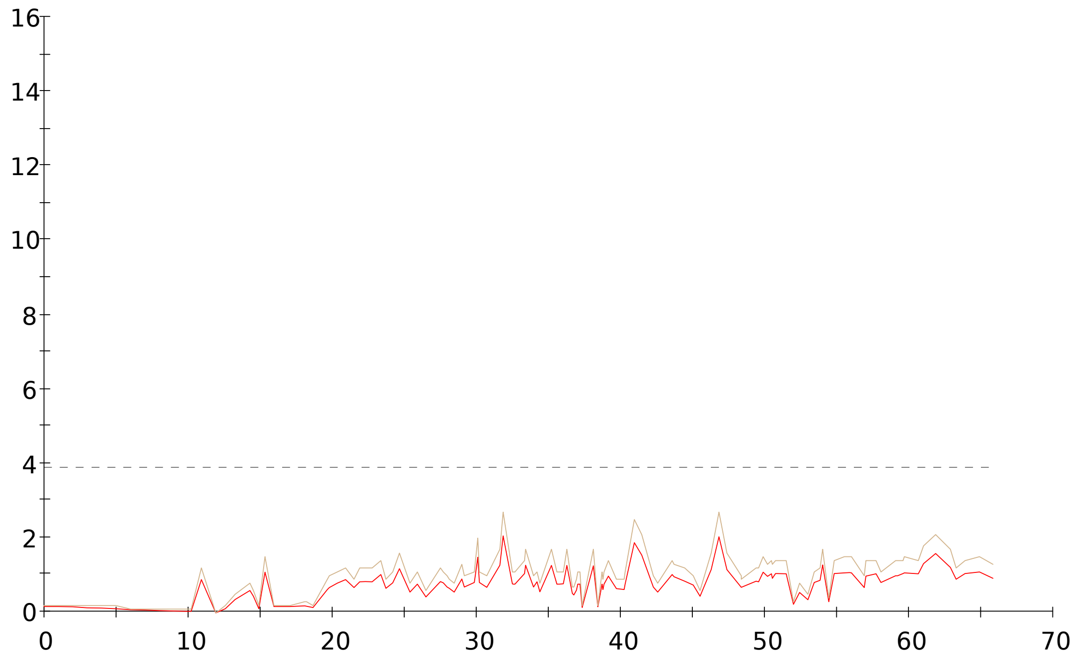

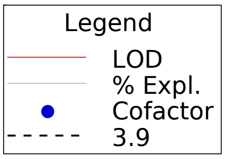

Group 6

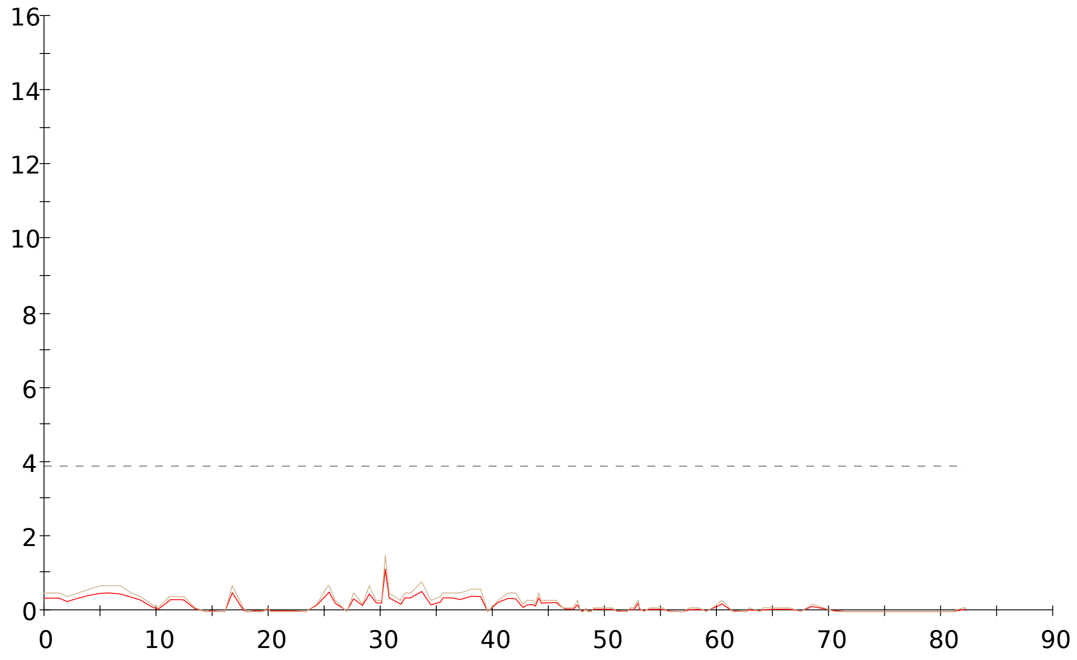

Group 7

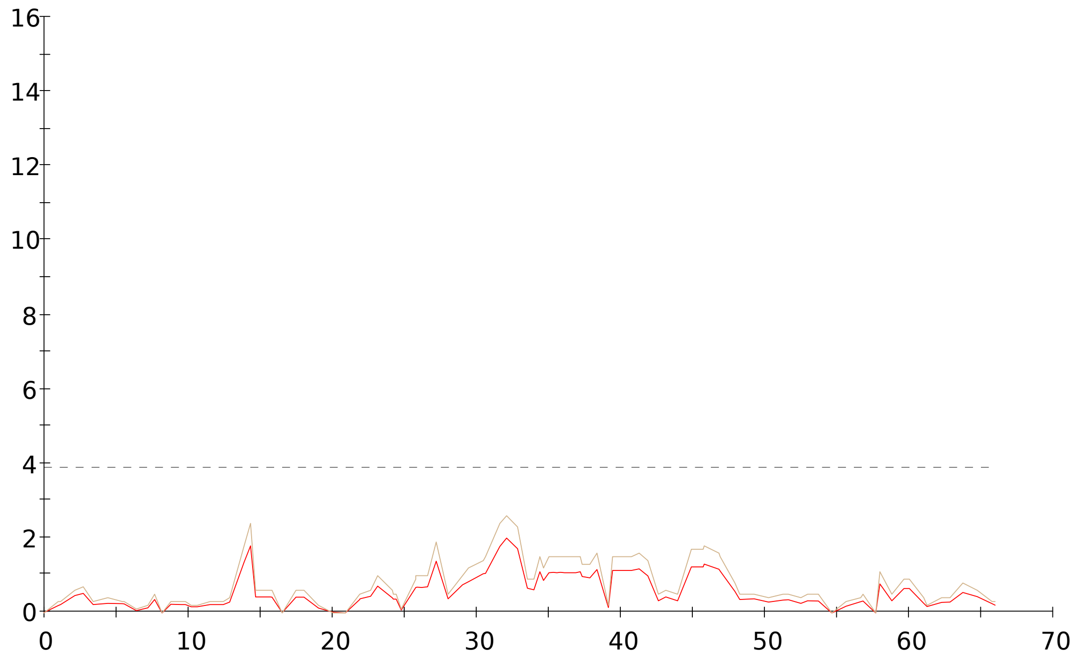

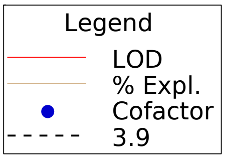

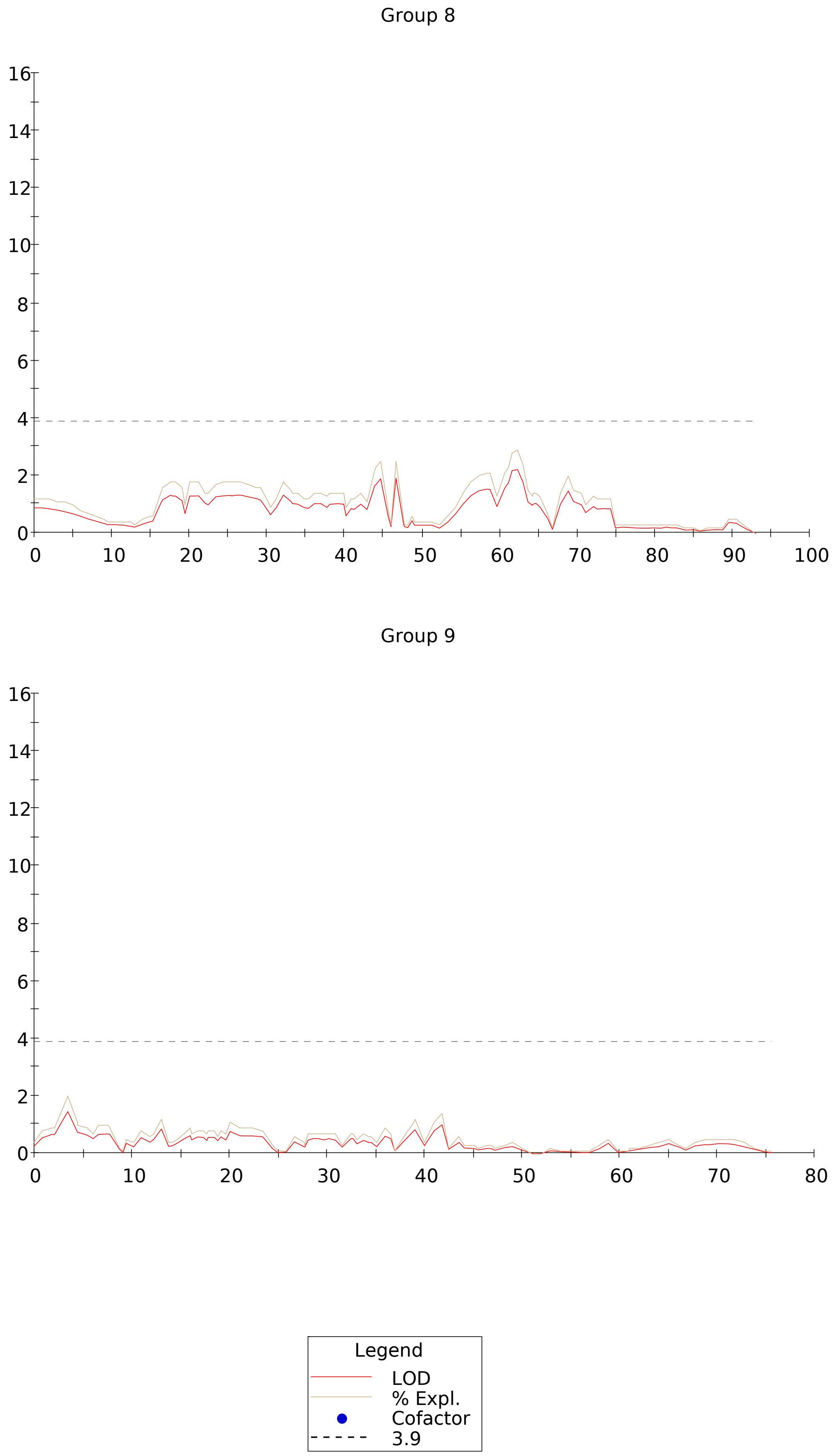
Group 8
16
14
12
10
8
6
4
2
0
0
10
20
30
40
50
60
70
80
90
100
Group 9
16
14
12
10
8
6
4
2
0
0
10
20
30
40
50
60
70
80
Legend
LOD
% Expl.
Cofactor
3.9

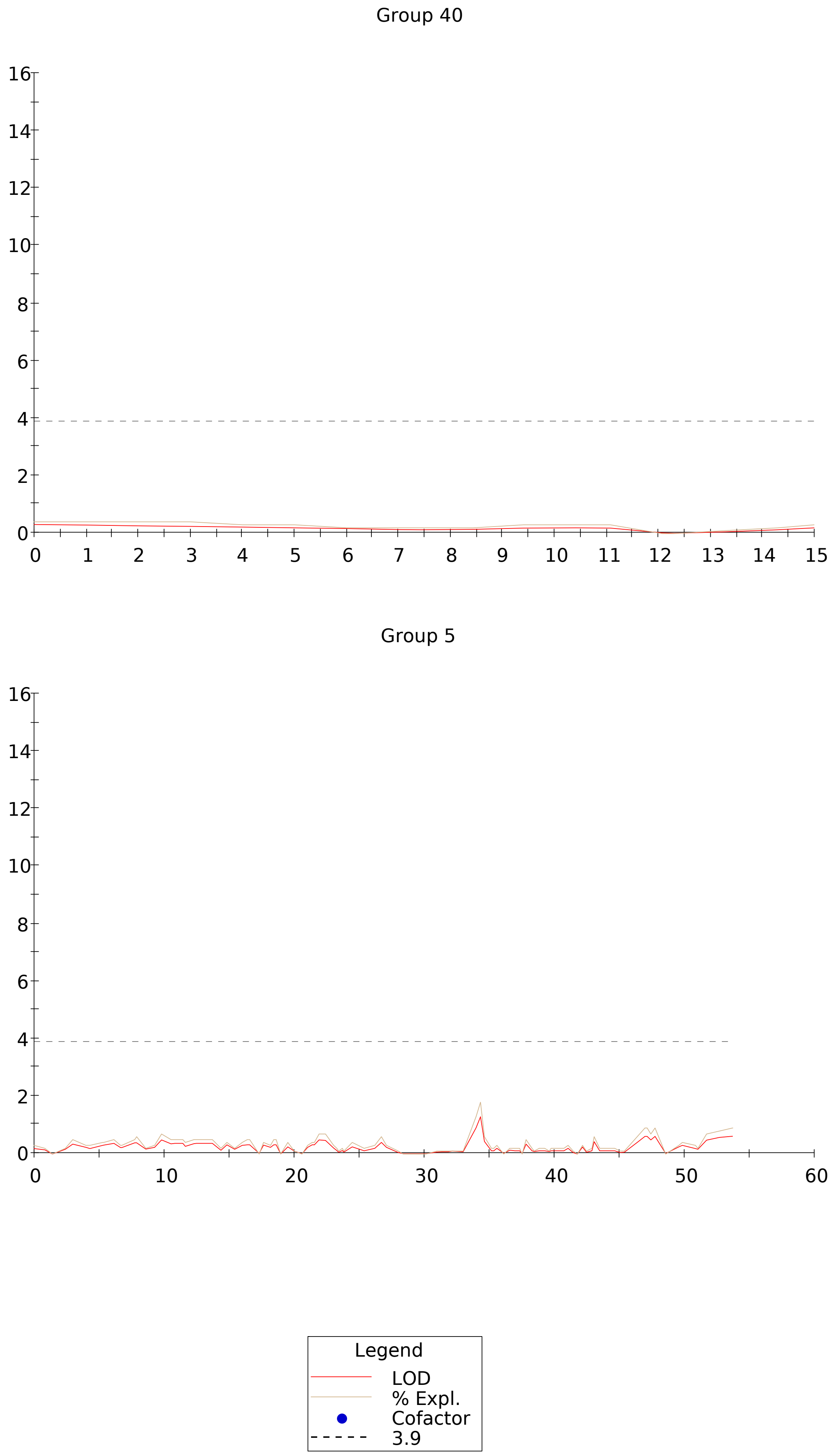
Group 40
16
14
12
10
8
6
4
2
0
0 1 2 3 4 5 6 7 8 9 10 11 12 13 14 15
Group 5
16
14
12
10
8
6
4
2
0
0 10 20 30 40 50 60
Legend
LOD
% Expl.
Cofactor
3.9
