# Supplementary material for: A High-Density Linkage Map of the Forage Grass Eragrostis curvula and Localization of the Diplospory Locus
Source: Front Plant Sci. 2019 Jul 12;10:918. doi: 10.3389/fpls.2019.00918 (PMC6640543; doi:10.3389/fpls.2019.00918)
Supplement: TABLE S1 — RAPD and SSR primers and AFLP adaptors, pre-amplification and selective primer sequences used to construct the E. curvula genetic maps. [file Table_1.DOCX]

**Supplementary Table S1:** RAPD and SSR primers and AFLP adaptors, pre-amplification and selective primer sequences used to construct the *E. curvula* genetic maps.

| **Nombre** | **Secuencia 5´- 3´** |
| --- | --- |
| RAPD primer 232 | CGGTGACATC |
| RAPD primer 245 | CGCGTGCCAG |
| RAPD primer 304 | AGTCCTCGCC |
| RAPD primer 331 | AGTCCTCGCC |
| SSR primer 10159 | F: TAGCCAGATGACCTCCAC  R: CCTGCTCTCCTCCGAC |
| SSR primer 13665 | F: TGCGCTGGACCTCTACTA  R: ACTGCTTCAGCCTCATCTT |
| SSR primer 13776 | F: TCCAACTCATCAACCAGTAA  R: GTAGCTCTTGCCGAACC |
| SSR primer 15838 | F: AGTGGAGGAAGATGTAGCC  R: ACAGAGTTGAAGGAGCAGAG |
| SSR primer 18079 | F: GACAGGACCCCTCTTCC  R: AGGACTCCCAGCTCAGAT |
| SSR primer 20053 | F: CCACCAACCAATTATCCTAA  R: GATCTCGCGACAACCC |
| SSR primer 20164 | F: GAACTACCTGCAGATCAAGG  R: TGCACTTTGTTGTCATCTTC |
| SSR primer 27968 | F: ACTTGCAGAAATCACAAAGG  R: GGATCGTGTTGATTGAAGTT |
| SSR primer 30246 | F: TCTTCTTCAGCCCCTTG  R: ACGTCGTCGTCATCCTC |
| *Pst*I adapter | GAC TGC GTA GGT GCA  CCT ACG CAG TCT ACG AG |
| *Mse*I adapter | GAC GAT GAG TCC TGA G  ATG AGT CCT GAG TA |
| *Pst*I +1 pre amplification primer | GAC TGC GTA CAT GCA GA |
| *Mse*I +1 pre amplification primer | GAT GAG TCC TGA GTA AA |
| *Pst*I +3 P36 selective amplification primer | GAC TGC GTA CAT GCA GACC |
| *Pst*I +3 P37 selective amplification primer | GAC TGC GTA CAT GCA GACG |
| *Pst*I +3 P40 selective amplification primer | GAC TGC GTA CAT GCA GAGC |
| *Pst*I +3 P41 selective amplification primer | GAC TGC GTA CAT GCA GAGG |
| *Mse*I +3 M31 selective amplification primer | GAT GAG TCC TGA GTA AAAA |
| *Mse*I +3 M38 selective amplification primer | GAT GAG TCC TGA GTA AACT |
| *Mse*I +3 M39 selective amplification primer | GAT GAG TCC TGA GTA AAGA |
| *Mse*I +3 M43 selective amplification primer | GAT GAG TCC TGA GTA AATA |
| *Mse*I +3 M45 selective amplification primer | GAT GAG TCC TGA GTA AATG |
