# Supplementary material for: A High-Density Linkage Map of the Forage Grass Eragrostis curvula and Localization of the Diplospory Locus
Source: Front Plant Sci. 2019 Jul 12;10:918. doi: 10.3389/fpls.2019.00918 (PMC6640543; doi:10.3389/fpls.2019.00918)
Supplement: TABLE S2 — Number of reads, length and coverage (average genome size of 1,250 Mb) of the GBS library from E. curvula used for SNP calling. Barcodes for each sample are indicated. [file Table_2.DOCX]

**Supplementary Table S2**. Number of reads, length and coverage (average genome size of 1,250 Mb) of the GBS library from *E. curvula* used for SNP calling. Barcodes for each sample are indicated.

| **Sample Name** | **Number of reads** | **Bases** | **coverage over genome (1,250Mb)** | **Barcodes** |
| --- | --- | --- | --- | --- |
| OTA-S | 8686226 | 96302141 | 0.0770417 | TTCTC - CGCCTTAT - TAACGA - TTCCTGGA |
| Don Walter | 6032130 | 85668723 | 0.0685350 | CTCC - GTCGATT - TACAT – TCTGTGA |
| Z002 | 7384611 | 93448324 | 0.0747587 | GCTTA |
| Z007 | 4588128 | 79385667 | 0.0635085 | AACGCCT |
| Z008 | 3290499 | 64043291 | 0.0512346 | AGGC |
| Z009 | 2192540 | 48372739 | 0.0386982 | TCGTT |
| Z011 | 5041626 | 78367157 | 0.0626937 | TGGCTA |
| Z012 | 3413555 | 62471714 | 0.0499774 | TGCTGGA |
| Z016 | 6421475 | 84386552 | 0.0675092 | TGCA |
| Z017 | 2405340 | 55402434 | 0.0443219 | AGCCC |
| Z020 | 5204427 | 79505163 | 0.0636041 | CTTCCA |
| Z021 | 2389801 | 51051466 | 0.0408412 | AATATGC |
| Z024 | 2665440 | 55271073 | 0.0442169 | GATC |
| Z025 | 4048032 | 71402419 | 0.0571219 | ACCTAA |
| Z028 | 2774892 | 56949093 | 0.0455593 | ACGTGTT |
| Z030 | 3946910 | 67310291 | 0.0538482 | AACCGAGA |
| Z031 | 3569332 | 64063151 | 0.0512505 | ACTA |
| Z032 | 2796563 | 58818606 | 0.0470549 | GTATT |
| Z033 | 3610063 | 66703303 | 0.0533626 | GAGATA |
| Z034 | 2490973 | 53530015 | 0.0428240 | ACGACTAC |
| Z039 | 2858932 | 57459187 | 0.0459673 | TCAC |
| Z056 | 2758106 | 54898642 | 0.0439189 | ATATGT |
| Z057 | 1771737 | 47841938 | 0.0382736 | ATTAATT |
| Z100 | 4183334 | 70607085 | 0.0564857 | ACAGGGAA |
| Z103 | 5706918 | 83505023 | 0.0668040 | CAGA |
| Z105 | 3732574 | 67924952 | 0.0543400 | CTGTA |
| Z109 | 2496781 | 56375384 | 0.0451003 | ATGCCT |
| Z111 | 3362015 | 65290408 | 0.0522323 | GGTGT |
| Z112 | 2953192 | 59059757 | 0.0472478 | AGGAT |
| Z113 | 3223946 | 63462614 | 0.0507701 | ATCGTA |
| Z115 | 2625251 | 56673112 | 0.0453385 | ATTGGAT |
| Z116 | 43 | 2300 | 0.0000018 | ACGTGGTA |
| Z119 | 3182001 | 58137442 | 0.0465100 | AACT |
| Z122 | 3793850 | 66734225 | 0.0533874 | ACCGT |
| Z128 | 2097744 | 53189133 | 0.0425513 | TATTTTT |
| Z131 | 2389376 | 52184428 | 0.0417475 | TAGCATGC |
| Z138 | 7899592 | 88710417 | 0.0709683 | ATTGA |
| Z140 | 5244109 | 76257866 | 0.0610063 | CATCGT |
| Z146 | 2277674 | 52499198 | 0.0419994 | CATAAGT |
| Z154 | 141 | 7843 | 0.0000063 | CCATGGGT |
| Z155 | 5150415 | 77459966 | 0.0619680 | GCGT |
| Z158 | 5495090 | 74397819 | 0.0595183 | GTAA |
| Z160 | 4353305 | 74562985 | 0.0596504 | CTTGCTT |
| Z162 | 3211307 | 59640185 | 0.0477121 | AGTGGA |
| Z167 | 4570508 | 72282386 | 0.0578259 | CATCT |
| Z171 | 4358408 | 72420309 | 0.0579362 | CGCGGT |
| Z174 | 2940174 | 60183907 | 0.0481471 | CGCTGAT |
| Z191 | 4161339 | 70808221 | 0.0566466 | CGCGGAGA |
| Z194 | 3944315 | 72536068 | 0.0580289 | TGCGA |
| Z195 | 3686441 | 67622753 | 0.0540982 | GGTTGT |
| Z197 | 2686593 | 59686691 | 0.0477494 | ATGAAAC |
| Z198 | 3980163 | 73468911 | 0.0587751 | TAGGCCAT |
| Z203 | 3047491 | 62246625 | 0.0497973 | CCTAC |
| Z205 | 4090934 | 73436653 | 0.0587493 | CTATTA |
| Z206 | 5595417 | 81710657 | 0.0653685 | CGGTAGA |
| Z207 | 3594271 | 66766676 | 0.0534133 | CGTGTGGT |
| Z208 | 4621284 | 74193184 | 0.0593545 | CGAT |
| Z209 | 4749631 | 79372014 | 0.0634976 | CCAGCT |
| Z210 | 2393924 | 57468554 | 0.0459748 | AAAAGTT |
| Z211 | 5109761 | 79165108 | 0.0633321 | TGCAAGGA |
| Z212 | 3842303 | 69380082 | 0.0555041 | GAGGA |
| Z213 | 4610694 | 75581028 | 0.0604648 | GCCAGT |
| Z214 | 5633506 | 83532422 | 0.0668259 | CTACGGA |
| Z215 | 3118270 | 61964249 | 0.0495714 | GCTGTGGA |
| Z216 | 3666689 | 68932120 | 0.0551457 | CGCTT |
| Z217 | 437881 | 18511153 | 0.0148089 | TTCAGA |
| Z218 | 6347847 | 84840536 | 0.0678724 | GAATTCA |
| Z219 | 6789993 | 87989046 | 0.0703912 | TGGTACGT |
| Z220 | 4281545 | 73754554 | 0.0590036 | GGAAC |
| Z221 | 4158009 | 67709438 | 0.0541676 | GGAAGA |
| Z222 | 3853240 | 68031641 | 0.0544253 | GCGGAAT |
| Z223 | 310 | 15123 | 0.0000121 | GGATTGGT |
| Z224 | 3281913 | 66014094 | 0.0528113 | TCACC |
| Z225 | 2868647 | 62083088 | 0.0496665 | TAGGAA |
| Z226 | 2988142 | 61808129 | 0.0494465 | GAACTTC |
| Z229 | 3465220 | 65469815 | 0.0523759 | TCTCAGTC |
| Z237 | 5534614 | 78628033 | 0.0629024 | GTCAA |
| Z251 | 3481431 | 64853943 | 0.0518832 | GTACTT |
| Z252 | 1735 | 93014 | 0.0000744 | TAGCGGA |
| Z258 | 3692875 | 67866726 | 0.0542934 | GTGAGGGT |
| Z259 | 3390186 | 66991778 | 0.0535934 | CTAGC |
| Z260 | 3739979 | 69460685 | 0.0555685 | GCTCTA |
| Z262 | 3784315 | 71015867 | 0.0568127 | GGACCTA |
| Z263 | 5520355 | 83011046 | 0.0664088 | CCGGATAT |
| Z266 | 2407760 | 56759379 | 0.0454075 | TAATA |
| Z268 | 3777322 | 67925757 | 0.0543406 | GTTGAA |
| Z269 | 2905223 | 61593101 | 0.0492745 | TCGAAGA |
| Z271 | 3430260 | 66868799 | 0.0534950 | TATCGGGA |
| Total | 328261542 | 5683418629 | 4.5467349 |  |
