# Supplementary material for: A High-Density Linkage Map of the Forage Grass Eragrostis curvula and Localization of the Diplospory Locus
Source: Front Plant Sci. 2019 Jul 12;10:918. doi: 10.3389/fpls.2019.00918 (PMC6640543; doi:10.3389/fpls.2019.00918)
Supplement: TABLE S5 — Synteny between the E. curvula sexual genotype OTA-S linkage groups and the physical map of O. thomaeum. [file Table_5.docx]

| **OTA**  **LG** | ***O. thomaeun* chromosomes** | | | | | | | | | | |
| --- | --- | --- | --- | --- | --- | --- | --- | --- | --- | --- | --- |
|  | **1** | **2** | **3** | **4** | **5** | **6** | **7** | **8** | **9** | **10** | **Others** |
| **1** |  |  |  | 17 |  |  | 1 |  |  |  |  |
| **2** |  |  | 1 | 9 |  |  |  | 1 |  |  |  |
| **3** |  |  |  | 2 |  | 12 | 1 |  |  |  | contig_108 |
| **4** | 1 |  | 2 | 44 | 2 | 2 | 1 | 1 |  |  |  |
| **5** | 1 | 29 |  |  | 1 | 1 |  |  | 1 |  |  |
| **6** | 15 |  | 1 | 2 | 2 | 2 |  |  |  |  |  |
| **7** |  |  |  |  |  | 1 | 14 |  |  |  |  |
| **8** |  | 2 | 2 | 2 | 19 | 1 |  |  |  |  |  |
| **9** |  |  |  |  |  |  | 2 |  | 15 |  |  |
| **10** |  | 1 | 14 |  | 2 |  | 2 | 1 |  | 1 |  |
| **11** | 14 | 2 |  |  | 2 |  |  | 2 |  |  |  |
| **12** |  | 14 | 2 | 1 |  | 1 | 1 |  |  | 1 |  |
| **13** | 1 | 13 | 1 |  | 1 |  | 1 |  |  | 1 |  |
| **14** |  |  | 1 | 19 | 2 |  | 1 |  |  | 1 |  |
| **15** | 1 |  | 12 | 1 | 1 |  |  |  |  | 2 |  |
| **16** | 3 | 2 | 3 | 1 |  |  |  | 7 |  |  |  |
| **17** | 12 |  |  |  |  |  |  | 1 |  |  |  |
| **18** |  |  | 8 |  |  |  |  |  |  |  | contig_2 |
| **19** |  |  | 12 |  |  |  |  |  |  |  |  |
| **20** |  | 2 |  |  |  | 11 |  |  |  |  | contig_89 |
| **21** |  |  |  |  | 1 |  | 11 |  | 1 | 1 |  |
| **22** |  |  | 8 | 1 |  |  |  |  |  |  |  |
| **23** | 1 | 2 |  |  |  |  |  |  | 6 |  |  |
| **24** |  | 1 |  | 1 |  |  |  | 2 |  | 1 |  |
| **25** |  |  |  |  |  |  |  | 5 |  |  |  |
| **26** |  |  | 1 |  | 8 |  |  |  |  |  |  |
| **27** |  |  |  |  |  |  |  |  |  | 5 |  |
| **28** |  |  | 2 |  |  |  |  |  |  |  |  |
| **29** |  |  |  |  | 1 |  |  |  |  |  |  |
| **30** |  |  |  |  |  |  |  |  | 2 |  | contig_89 |
| **31** |  |  |  |  |  |  | 3 | 1 |  |  |  |
| **32** | 1 |  | 2 |  | 1 |  |  |  |  |  |  |
| **33** |  |  |  |  |  |  |  |  |  | 4 |  |
| **34** |  | 2 |  |  |  |  |  |  |  |  |  |
| **35** |  |  |  |  |  |  |  |  |  |  | Without homology |
| **36** |  |  |  |  |  |  |  |  |  |  | Without homology |
| **37** |  |  |  |  |  |  |  |  |  |  | Without homology |
| **38** |  |  |  |  |  |  |  |  |  |  | Without homology |
| **39** |  |  |  |  |  |  |  |  |  |  | Without homology |
| **40** |  |  |  | 2 |  |  |  |  |  |  |  |
