# Supplementary material for: A High-Density Linkage Map of the Forage Grass Eragrostis curvula and Localization of the Diplospory Locus
Source: Front Plant Sci. 2019 Jul 12;10:918. doi: 10.3389/fpls.2019.00918 (PMC6640543; doi:10.3389/fpls.2019.00918)
Supplement: TABLE S6 — Synteny between the E. curvula facultative apomictic cultivar Don Walter linkage groups and the physical map of O. thomaeum. [file Table_6.docx]

| **DW**  **LG** | ***O. thomaeun* chromosomes** | | | | | | | | | | |
| --- | --- | --- | --- | --- | --- | --- | --- | --- | --- | --- | --- |
|  | **1** | **2** | **3** | **4** | **5** | **6** | **7** | **8** | **9** | **10** | **Others** |
| **1** | 48 | 1 | 1 |  |  | 2 |  |  | 1 | 1 | contig_2 |
| **2** |  | 43 |  | 2 |  |  | 1 | 1 | 1 | 6 |  |
| **3** |  |  | 1 |  | 20 | 1 | 1 | 1 |  |  |  |
| **4** | 51 | 4 |  |  | 1 |  | 2 |  |  | 1 |  |
| **5** | 3 |  | 46 |  | 1 | 1 | 1 |  |  | 1 | contig_23, contig_66 |
| **6** |  |  | 37 |  | 1 | 2 | 2 |  |  | 3 |  |
| **7** | 4 | 2 | 2 | 37 |  |  |  | 1 |  | 2 |  |
| **8** |  |  | 2 | 3 | 2 |  | 19 | 8 |  |  |  |
| **9** | 3 | 18 |  |  | 2 | 1 | 1 |  |  |  | contig_10(2),contig_148 |
| **10** | 1 | 2 | 1 |  | 1 | 3 | 17 |  | 1 |  |  |
| **11** |  |  | 3 | 2 | 23 |  | 2 | 1 |  |  |  |
| **12** |  | 22 |  |  |  |  | 1 |  |  |  | contig_1 |
| **13** |  | 5 |  |  |  |  |  |  | 2 | 7 |  |
| **14** | 18 |  | 1 | 1 |  |  |  |  | 2 |  | conitg_57 |
| **15** | 2 | 3 |  | 1 |  | 2 | 1 |  | 15 |  |  |
| **16** | 1 |  |  |  |  | 20 | 1 |  |  | 1 |  |
| **17** |  | 2 |  |  |  |  |  |  | 18 |  | contig_60 |
| **18** |  | 1 | 1 | 1 |  |  | 14 |  |  |  |  |
| **19** |  | 1 |  | 19 | 2 | 3 |  |  |  |  |  |
| **20** | 1 | 1 | 1 |  | 1 | 17 | 1 |  |  |  |  |
| **21** |  |  |  |  | 1 |  |  | 12 |  | 2 |  |
| **22** | 20 | 2 |  | 1 |  |  |  | 1 |  |  |  |
| **23** |  | 2 | 1 | 20 |  |  | 1 |  |  |  |  |
| **24** | 1 |  |  | 17 |  |  |  |  |  | 10 |  |
| **25** |  |  | 18 |  | 2 |  |  |  |  |  |  |
| **26** | 1 |  |  |  | 15 |  |  |  |  |  |  |
| **27** |  |  |  |  |  |  | 1 |  | 14 |  | contig_89 |
| **28** | 1 |  |  |  |  | 11 |  |  |  |  | contig_91 |
| **29** |  |  | 13 |  |  |  |  |  |  |  |  |
| **30** |  |  |  |  | 11 |  |  |  | 1 |  |  |
| **31** |  |  |  | 15 |  |  |  |  |  |  |  |
| **32** |  |  |  |  |  | 1 | 3 |  |  | 1 |  |
| **33** |  |  |  |  |  | 6 |  | 2 |  |  |  |
| **34** |  | 3 | 2 |  |  |  |  |  |  |  |  |
| **35** |  | 2 | 2 |  |  |  |  |  | 1 | 2 |  |
| **36** |  | 4 |  |  |  |  |  |  |  |  | contig_1 |
| **37** |  |  |  |  |  |  |  | 2 |  |  |  |
| **38** |  |  |  |  |  |  | 1 |  |  |  |  |
| **39** |  |  |  |  |  |  |  |  |  | 4 |  |
| **40** |  |  | 1 | 3 |  |  |  |  | 3 |  |  |
